# Synergistic backbone-side chain design *via* noncovalent interactions for advanced high-temperature proton exchange membranes

**DOI:** 10.1039/d6sc04018g

**Published:** 2026-08-11

**Authors:** Qian Wang, Wenzhe Zhao, Zhejing Zhang, Yang Wu, Jingshuai Yang

**Affiliations:** a Department of Chemistry, College of Sciences, Northeastern University Shenyang 110819 China yjs@mail.neu.edu.cn; b College of Chemistry, Liaoning University Shenyang 110036 Liaoning China

## Abstract

High-temperature proton exchange membrane (HT-PEM) fuel cells are essential for efficient hydrogen energy conversion, yet their performance is often limited by insufficient proton transport and poor phosphoric acid (PA) retention of HT-PEMs. Current strategies mainly rely on covalent structural modification, with limited focus on intermolecular interactions. Here, we propose a molecular design that regulates non-covalent interactions to simultaneously enhance conductivity, chemical stability, and PA retention. Poly(arylene alkylene) membranes were synthesized by incorporating dibenzo-18-crown-6 units into an aromatic backbone and flexible alkyl side chains bearing quaternary ammonium or imidazolium groups. The crown ether units introduce dipole interactions and additional PA binding sites, while the flexible side chains enable dynamic coupling with the backbone. Methyl-substituted imidazolium groups further strengthen PA binding through improved charge delocalization. This synergistic design promotes well-defined microphase separation and forms a stable proton transport network. The optimized membrane (PTC_0.3_H-DMI) exhibits a proton conductivity of 65.3 mS cm^−1^ at 180 °C and a peak power density of 1152 mW cm^−2^ under anhydrous conditions. It also maintained stable voltage output during long-term operation under 200 and 400 mA cm^−2^ at 160 °C. This work highlights the importance of non-covalent interactions (especially dipole coupling and hydrogen bonding) and provides a practical strategy for designing advanced HT-PEMs.

## Abstract 2

A molecular design that regulates non-covalent interactions to simultaneously enhance conductivity, chemical stability, and PA retention is proposed for high-performance high temperature proton exchange membranes.

## Graphical abstract

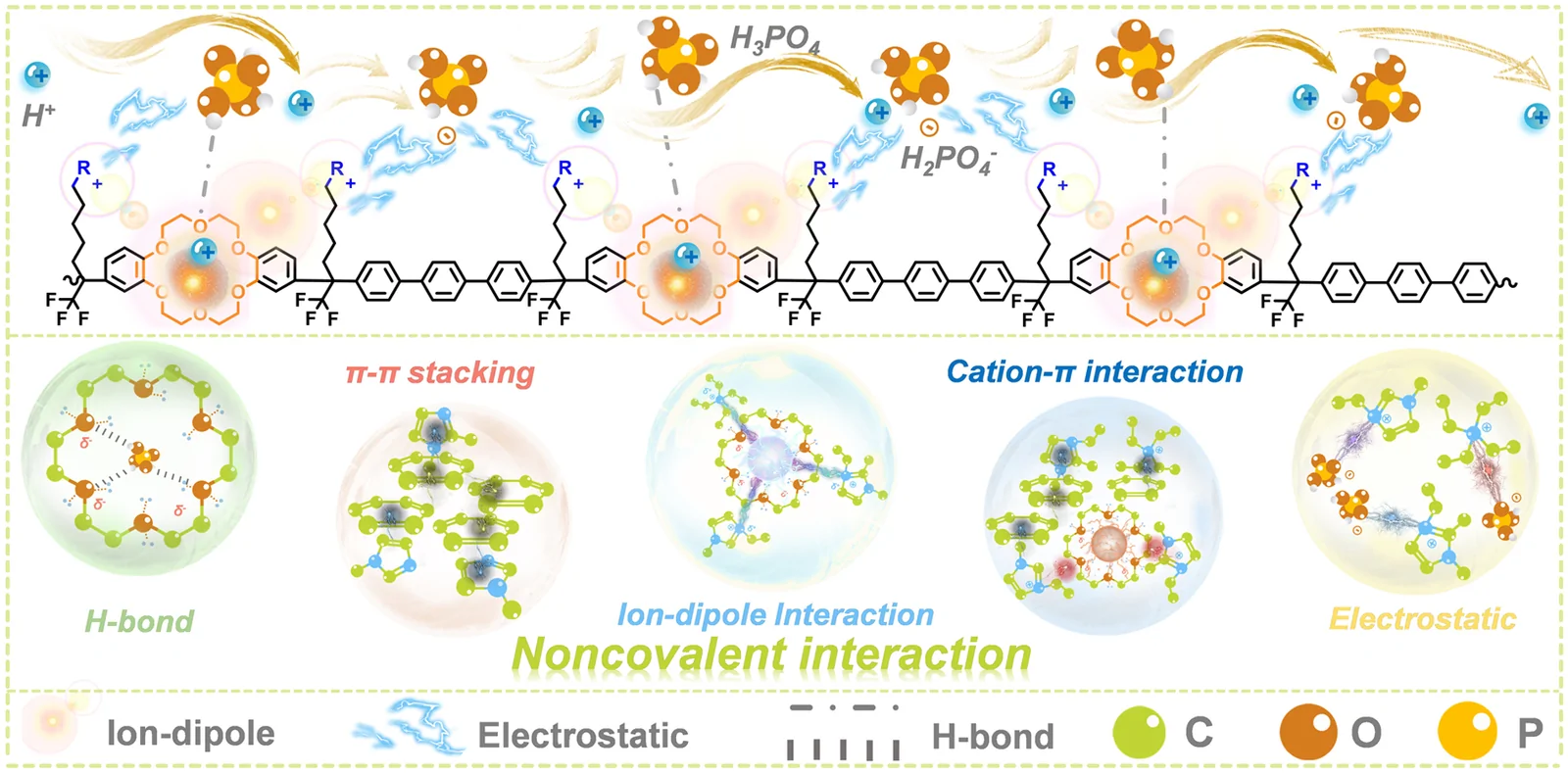

## 1 Introduction

Hydrogen proton exchange membrane fuel cells are a key technology for efficient hydrogen utilization, yet their large-scale deployment is still limited by challenges in performance and durability.^1–3^ Compared with conventional low-temperature systems based on PFSA membranes, high-temperature proton exchange membrane fuel cells (HT-PEMFCs) offer distinct advantages,^4,5^ including simplified water management,^6,7^ faster reaction kinetics,^8,9^ and improved tolerance to fuel impurities.^5,10^ At the heart of HT-PEMFCs, the high temperature proton exchange membrane (HT-PEM) plays a critical role by enabling proton transport while preventing gas crossover,^2,11,12^ thereby directly determining power output and long-term stability. Although phosphoric acid (PA) doped polybenzimidazole (PBI) membranes are widely regarded as benchmark materials,^13–15^ they still suffer from several intrinsic limitations. In particular, PA leaching remains a major degradation pathway due to the relatively weak and reversible interactions between PA and imidazole groups.^12,16,17^ In addition, residual reactive end groups can trigger hydrolytic and oxidative degradation, while the rigid aromatic backbone leads to poor solubility and limited processability.^2,18,19^ These issues highlight the urgent need for advanced HT-PEMs with improved conductivity, stability, and processability.

To address these challenges, polymers synthesized *via* superacid-catalyzed polymerization of fully aromatic frameworks with basic functional groups have attracted increasing attention.^20–22^ Among them, poly(arylene piperidine)s (PAPs) exhibit strong PA affinity and serve as versatile platforms for further structural design.^21,23^ Recently, the incorporation of π-conjugated heterocycles such as pyridine,^24^ imidazole,^25^ and thiazole,^26^ together with structural modification strategies including side-chain grafting,^27^ copolymerization,^23,28^ and branching,^29,30^ has proven highly effective in improving membrane performance. In particular, flexible alkyl side chains can promote microphase separation and the formation of continuous proton-conducting pathways,^4,31,32^ while also reducing backbone plasticization by spatially separating functional groups.^31^ Careful control of side-chain length is essential to balance conductivity, morphology, and mechanical stability, as demonstrated by high-performance systems with optimized spacer structures.^33^ Notably, imidazole-based functional groups show clear advantages over conventional quaternary ammonium groups due to their higher chemical stability and stronger interaction with PA.^25,34,35^ Their aromatic nature and electron delocalization enable efficient proton transport and enhanced acid retention,^36^ making them especially attractive for the design of high-performance HT-PEMs.

Despite these advances, the use of rigid all-aromatic backbones still imposes a trade-off between mechanical stability and functional group density, limiting the number of active sites available for proton transport.^22,24,37^ To overcome this limitation, integrating functional units directly into the backbone has emerged as an effective strategy.^38,39^ In this context, crown ether units such as dibenzo-18-crown-6 are particularly promising due to their strong interaction with PA, which enhances acid uptake and facilitates proton transport. Previous studies have demonstrated that incorporating DBC into polymer backbones can significantly improve membrane performance.^40–42^ As an example of our previous work,^40^ the PA doped poly(aryl pyridine) membranes with dibenzo-18-crown-6 segments delivered a peak power density of nearly 1200 mW cm^−2^ at 180 °C without humidification and backpressure, ranking among the highest values reported to date. However, efficient utilization of crown ether units requires sufficient mobility of functional groups to enable effective interactions. Introducing long and flexible side chains provides the necessary conformational freedom, allowing functional groups to dynamically interact with crown ether rings. This promotes ion–dipole interactions and supports the formation of efficient proton transport pathways,^43^ offering a robust strategy for constructing stable and high-performance HT-PEMs.

Building on this design concept, we developed a series of poly(arylene alkylene)s based on *p*-terphenyl, dibenzo-18-crown-6 (DBC) and 7-bromo-1,1,1-trifluoroheptan-2-one monomers, denoted as PTC_*n*_H, where *n* represents the molar fraction of DBC units incorporated into the copolymer backbone. By tuning the DBC content, a family of rigid-flexible PTC_*n*_H copolymers was obtained as membrane matrices. In this system, the crown ether units enhance hydrophilicity and microstructural organization while providing additional acid interaction sites and forming continuous proton transport pathways. Meanwhile, imidazolium groups were grafted onto the bromoalkyl side chains to create strong PA-binding sites that are spatially separated from the polymer backbone. The long and flexible side chains provide sufficient conformational freedom, enabling the terminal functional groups to form stable and directional dipole interactions with the crown ether rings. This integrated design enhances chemical stability and suppresses PA loss, while further enriching the multidimensional proton transport network.

A systematic comparison of different functional groups demonstrates that imidazolium-based systems outperform conventional quaternary ammonium counterparts in both stability and fuel cell performance. Additional improvements are achieved through methyl substitution on the imidazole ring (*i.e.*, C2-methyl-substituted dimethylimidazolium), which fine-tunes the electronic structure of the cation. Overall, this dual design strategy creates a strong synergy between backbone and side chains. Grafting imidazolium groups onto long alkyl side chains effectively reduces the plasticization effect of PA on the rigid backbone, thereby preserving dimensional and mechanical stability, while simultaneously providing strong acid-binding sites and stable proton-conducting centers. Compared with our previously reported fully aromatic pyridine-based DBC-containing membrane (*i.e.*, P(TP_91%_-co-CE_9%_))^40^ as summarized in Table S1, the present design introduces a fundamentally different molecular strategy by employing non-covalent interactions between the crown ether-containing backbone and flexible cationic side chains. Rather than serving only as a functional backbone component, the DBC unit acts as an interaction-regulating platform that dynamically controls PA distribution, chain organization, and proton transport pathways through ion–dipole interactions and hydrogen bonding as illustrated in Scheme 1. This backbone-side-chain cooperative strategy provides a new approach for constructing high-performance HT-PEMs by regulating intermolecular non-covalent interactions.

**Scheme 1:**
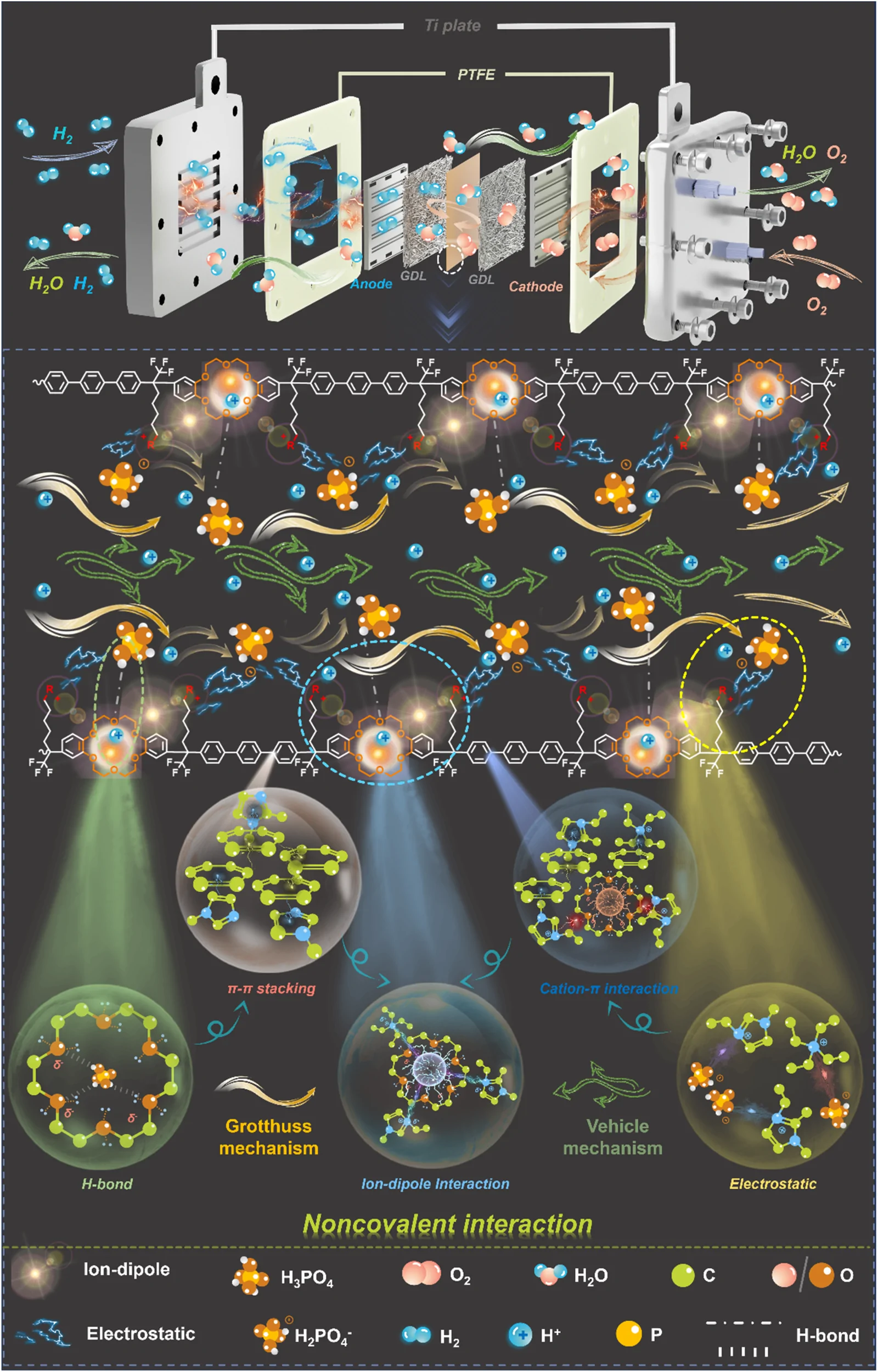
Schematic of the improvement strategies for PTC_*n*_H based HT-PEMs designed in this work, and the working principle in HT-PEMFC.

## 2 Results and discussion

### 2.1 Molecular design based on theoretical calculations

During fuel cell operation, reactive radicals generated from incomplete gas reactions can attack polymer chains and induce chemical degradation of HT-PEMs. Understanding this degradation mechanism is essential for rational molecular design.^35,44^ Due to their electrophilic nature, these radicals preferentially attack electron-rich regions along the polymer backbone. Density functional theory (DFT) calculations, including analysis of frontier molecular orbitals and electrostatic potential (ESP) distributions, provide an effective way to evaluate the relative reactivity of different functional groups. As shown in Fig. 1a, b, S1 and S2 (in SI), imidazolium-based groups exhibit relatively low HOMO energy levels, indicating improved resistance to oxidative degradation.^45^ More importantly, the presence of the imidazolium cation directs radical attack toward β-hydrogen atoms located away from the imidazole ring. Consistent with this, the calculated activation energy for β-H abstraction is lower than that for α-H abstraction (Fig. 1c and S3), confirming the preferred degradation pathway. Further analysis of imidazolium derivatives shows that methyl substitution plays a key role in stability. Although the unsubstituted imidazolium system exhibits a higher transition-state energy barrier, it also has a lower LUMO energy, making it more vulnerable to nucleophilic attack.^42,46^ In contrast, methyl substitution introduces hyperconjugation between C–H σ bonds and the aromatic π system, which stabilizes the transition state during hydrogen abstraction and improves overall resistance to degradation. Based on these results, dimethyl-substituted imidazolium is expected to provide enhanced stability. For comparison, non-cyclic quaternary ammonium groups were also included to evaluate the combined effects of stability and proton transport.

**Fig. 1:**
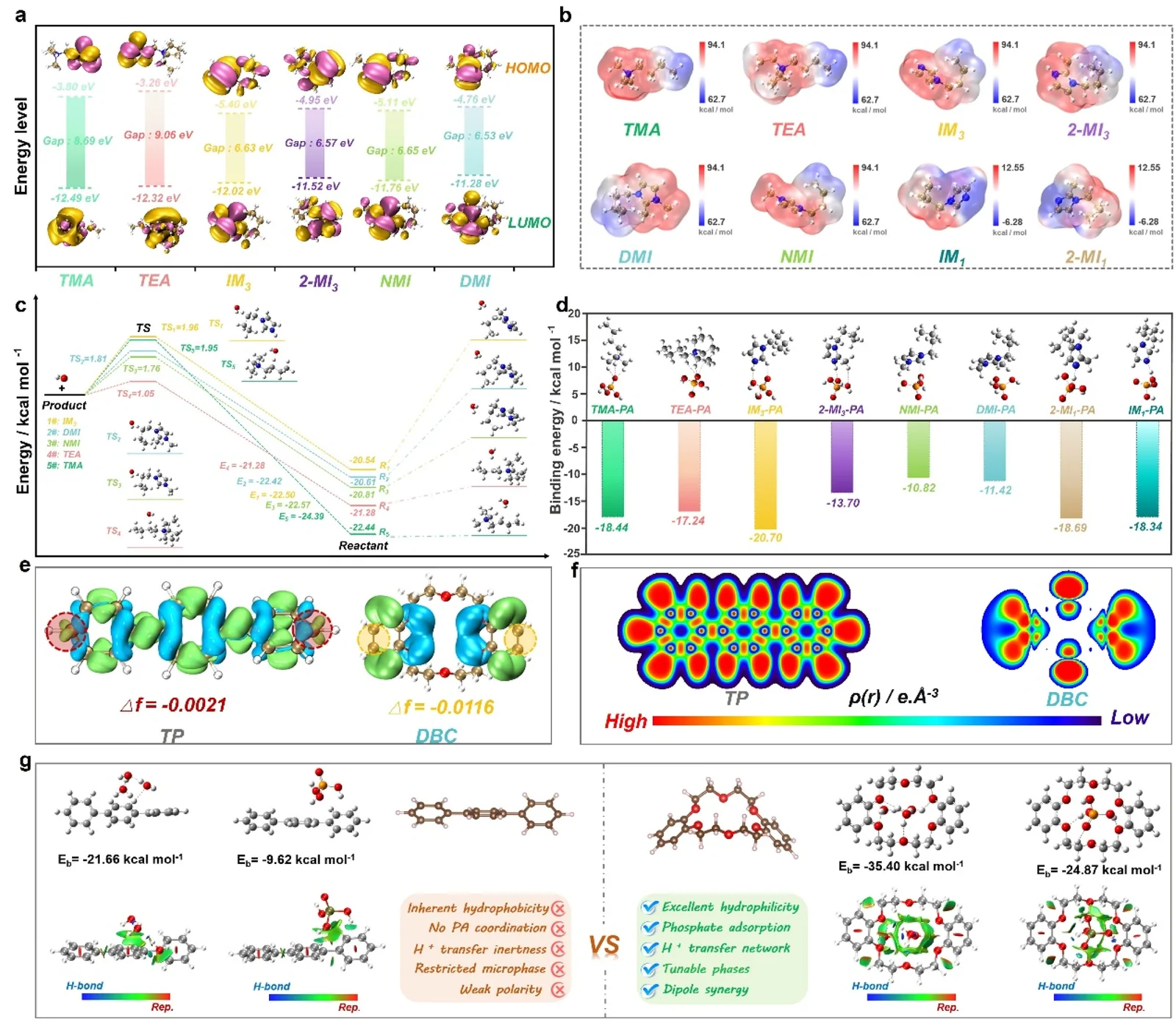
(a) Orbital energy levels, (b) ESP maps, (c) transition states for radical attack, and (d) PA binding energies of various imidazolium and QA groups; (e) dual descriptor isosurfaces and (f) electron localization function (ELF) of TP and DBC monomers; (g) structural and hydrophilic features of TP and DBC monomers.

Because proton conduction in HT-PEMs mainly relies on doped PA molecules, the interaction between functional groups and PA is another critical factor.^40,47^ Therefore, the PA binding energy (*E*_b_) of different species was calculated (Fig. 1d). Both imidazolium and quaternary ammonium groups show changes in *E*_b_ upon methyl substitution. Notably, dimethylimidazolium (DMI) exhibits a higher binding energy with PA than *N*-methylimidazolium (NMI), despite the electron-donating nature of methyl groups. This behavior can be explained by the strong electrostatic interaction between PA and the positively charged cation. In DMI, methyl substitution increases charge localization on the nitrogen atom and promotes a more favorable orientation of PA, leading to stronger interaction. In contrast, partial charge delocalization in NMI weakens this interaction. Based on these insights, TEA, NMI, and DMI were selected as representative side-chain functional groups for experimental validation.

Beyond functional group design, optimization of the polymer backbone is equally important. To fully utilize the properties of side-chain groups, the backbone structure must be carefully engineered.^28,45^ In this work, a crown ether unit of DBC was incorporated into a *p*-terphenyl-based backbone, and a flexible heptane linker was introduced to connect the functional segments. This design allows better control over chain packing and nanoscale morphology. A series of fluorinated poly(arylene alkylene) copolymers, including the homopolymer PTH derived from *p*-terphenyl and 7-bromo-1,1,1-trifluoroheptan-2-one, as well as DBC-containing copolymers PTC_*n*_H (*n* ≤ 0.5), were synthesized *via* superacid-catalyzed polymerization (Fig. S4). The DBC content was limited to ensure good processability and membrane-forming ability.

DFT results reveal several advantages of the DBC-containing backbone compared with the conventional terphenyl structure. First, DBC shows higher chemical reactivity during superelectrophilic polymerization reaction, as indicated by lower dual descriptor values and electron localization function (ELF) distributions (Fig. 1e and f). Second, DBC exhibits stronger interaction with PA, leading to higher adsorption capacity and additional proton transport pathways (Fig. 1g and S5). Third, the electron-rich crown ether structure can interact with side-chain cations through dipole interactions, which helps stabilize the functional groups and improve durability.^42^ In addition, the introduction of polar and flexible segments enhances solubility of copolymers in common organic solvents such as *N*,*N*-dimethylacetamide (DMAc) and 1-methyl-2-pyrrolidinone (NMP), enabling scalable membrane fabrication. For example, the maximum soluble amount in 1 mL of DMAc at 25 °C increased from 1.76 g for PTH to 2.87 g for PTC_0.5_H, quantitatively confirming the improved solubility after DBC incorporation (Fig. S6). Overall, this combined design, featuring a crown ether-containing aromatic backbone and long alkyl side chains grafted with basic functional groups, provides a rational strategy to simultaneously improve properties such as proton conduction, acid retention, and chemical stability of HT-PEMs.

### 2.2 Polymer structure and membrane morphology

The chemical structure of the target polymers was clearly confirmed by ^1^H NMR and FTIR spectroscopy. The ^1^H NMR spectra before and after quaternization, recorded in CDCl_3_ and DMSO-*d*_6_, are shown in Fig. 2a, b and S7. After introducing the DBC unit, two characteristic peaks appeared at 4.00 and 4.15 ppm, which are assigned to the methylene protons (–CH_2_–) in the crown ether ring.^40,44^ By comparing the integral areas of these signals with those of the characteristic methylene protons adjacent to the bromine atom (3.36 ppm), the actual DBC contents in the copolymers were calculated to be 13.75%, 29.63%, and 50.12% for PTC_0.1_H, PTC_0.3_H, and PTC_0.5_H, respectively. These values closely match the designed feed ratios, confirming the successful incorporation of DBC units into the copolymer backbone. The aromatic protons are located in the range of 7.3–8.0 ppm,^26^ while signals between 1.5 and 4.0 ppm correspond to the aliphatic methylene groups of the heptane side chains.^32^ After quaternization, a clear downfield shift of the α-methylene protons adjacent to the cationic center was observed, due to the electron-withdrawing effect of the positively charged nitrogen. For example, this signal appears at 4.00 ppm in PTH-DMI and shifts further to 4.06 ppm in PTH-NMI. This difference reflects the lower electron density in the NMI system, caused by fewer methyl substituents, which leads to stronger deshielding. Therefore, the chemical shift of these protons can be used as a sensitive indicator of the electronic effect of different functional groups. FTIR results further support these assignments, with characteristic peaks at 1250 cm^−1^ (C–O stretching of the crown ether) and 1580 cm^−1^ (C–N/C=N stretching after quaternization),^40^ confirming the successful incorporation of both backbone and side-chain functionalities.

**Fig. 2:**
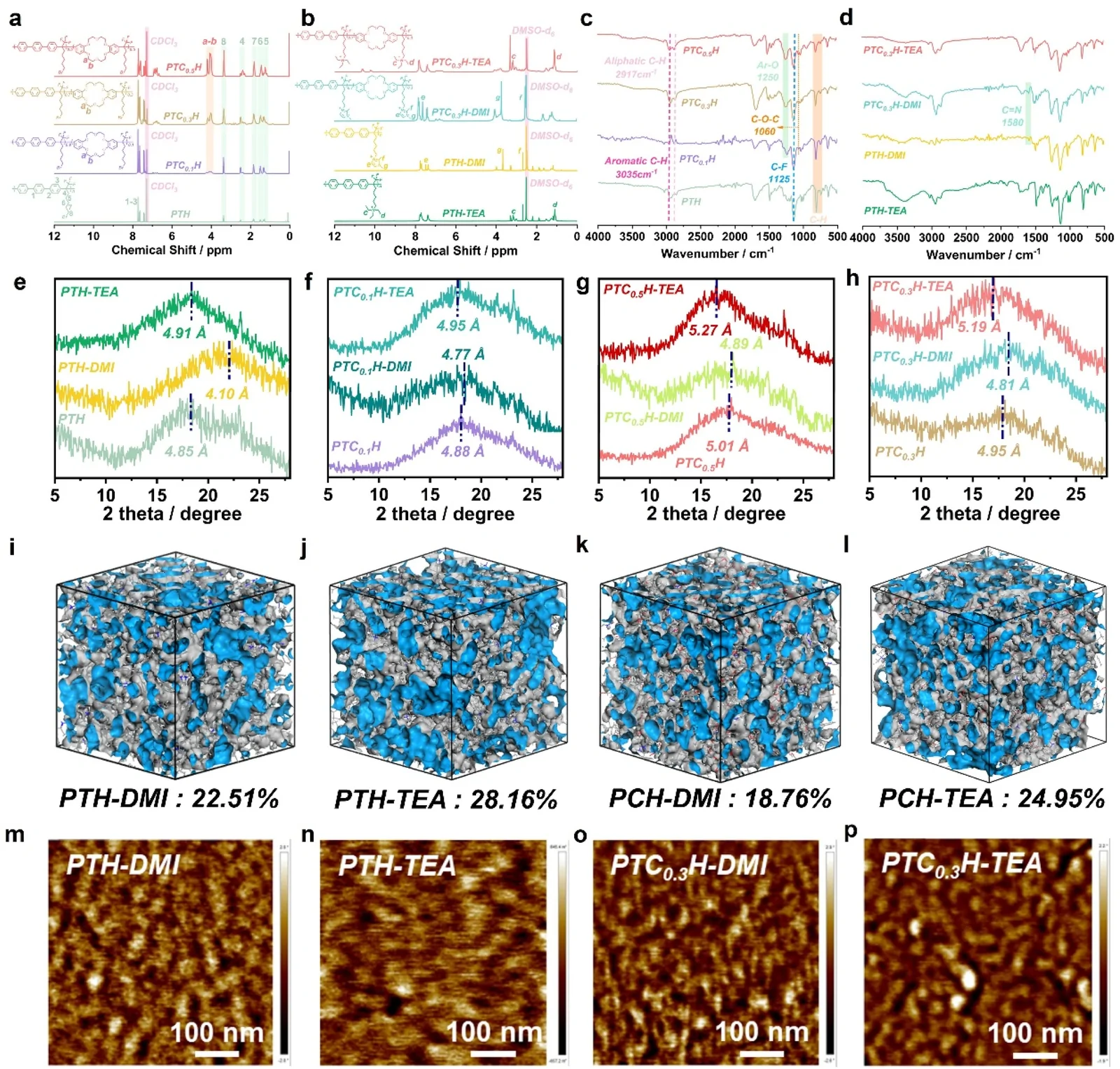
(a and b) ^1^H NMR and (c and d) FTIR spectra of PTC_*n*_H and their quaternized polymers. (e–h) XRD patterns, (i–l) MD simulations (accessible volume in blue), (m–p) AFM of various membranes.

Wide-angle X-ray diffraction (WXRD) was used to study chain packing behavior. The incorporation of the DBC unit, with its flexible ether linkages and macrocyclic structure, increases interchain spacing,^42^ as evidenced by the shift of diffraction peaks to lower angles (Fig. 2e–h and S10). During quaternization, two competing effects influence the microstructure: occupation of free volume by bulky cationic groups and electrostatic repulsion between charged chains. Their combined effect determines the final packing structure and transport properties.^30,48^ Molecular dynamics (MD) simulations (Fig. 2i–l and S11) further reveal the dynamic rearrangement of polymer chains, providing insight into the relationship between nanoscale structure and proton transport. A key feature of this system is the long and flexible heptane side chain, which behaves differently from rigid pyridine side-chain structures in P(TP_*x*%_-co-CE_*y*%_) reported previously.^40^ Due to its high flexibility, the side chain can partially fold into the DBC cavity, reducing effective free volume. Although the bulky DBC unit increases average interchain spacing, this effect is partially offset by cavity filling, geometric constraints, and enhanced intermolecular interactions. As a result, the fractional free volume (FFV) decreases from 22.39% for PTH to 17.65% for PCH, where PCH represents the corresponding homopolymer originated from DBC and 7-bromo-1,1,1-trifluoroheptan-2-one. After quaternization, the introduction of cationic groups increases FFV due to electrostatic repulsion and conformational disorder,^46^ reaching 22.51% for PTH-DMI and 28.16% for PTH-TEA. However, the increase is moderate in PTH-DMI because the planar imidazolium ring forms π–π interactions with the aromatic backbone, leading to more compact packing. In PCH-DMI, strong dipole interactions between the imidazolium cation and the crown ether oxygen atoms further restrict chain mobility, limiting FFV expansion. Overall, while tetramethylammonium cations provide higher FFV and may favor rapid proton transport, the DBC-based system introduces additional, directional proton transport pathways and improved structural stability, which are advantageous for long-term fuel cell operation.

The incorporation of DBC into the polymer backbone would reduce the effective conjugation length, as revealed by the π electron delocalization situation of TP and DBC (Fig. S12). This change is also reflected in the gradual lightening of membrane color with increasing DBC content of PTC_*n*_H membranes (Fig. S13). In contrast, the introduction of functional basic groups including TEA, DMI, and NMI enhances electronic interactions with the conjugated backbone, leading to a red shift and darker membrane appearance. These optical changes provide direct evidence for successful structural modification. As shown in Fig. S13, SEM images demonstrate that most membranes exhibit smooth and dense surfaces, indicating excellent membrane-forming capability.^25^ However, the membrane with the highest DBC content (PTC_0.5_H) displays a relatively rough surface morphology. Cross-sectional SEM images further confirm that these structural irregularities extend throughout the membrane thickness and its corresponding quaternized derivative (Fig. S14). In contrast, all other membranes display dense and smooth cross-sectional morphologies. This is probably attributed to the strong polarity difference between the relatively polar crown ether units and the nonpolar terphenyl backbone, which promotes phase separation and is unfavorable for gas barrier performance.^49^

Under high-temperature and low-humidity conditions, proton transport mainly follows the Grotthuss mechanism, which strongly depends on the formation of well-developed microphase-separated structures and continuous hydrogen-bonded networks.^21,50^ AFM phase images (Fig. 2m–p and S15) clearly show nanoscale phase separation, with hydrophobic domains appearing bright and hydrophilic domains appearing dark.^4,33^ The introduction of crown ether units and functional side chains increases the fraction of flexible and hydrophilic domains, indicating the formation of well-defined proton-conducting regions. This observation is consistent with water contact angle (Fig. S16) and water uptake (Fig. S17) measurements, which show improved hydrophilicity after DBC incorporation. TEM images of chloroplatinic acid-stained membranes (Fig. S18) further confirm the formation of proton-conducting domains. The dark regions correspond to areas rich in hydrophilic functional segments,^27,47^ which serve as proton transport sites. With increasing DBC content, these domains become more continuous and interconnected, indicating enhanced microphase separation and more efficient proton transport pathways. Overall, the combined results from SEM, AFM, and TEM demonstrate that the introduction of crown ether units and basic groups functionalized long alkyl side chains enables precise control over nanoscale morphology. This well-organized microphase-separated structure is essential for achieving high proton conductivity and stable performance in HT-PEMs.

### 2.3 Performance characterization and MD insights

PA uptake is a key parameter that directly reflects the proton conductivity of PA doped HT-PEMs. Under identical conditions, TEA-functionalized membranes show higher PA uptake than DMI- and NMI-functionalized counterparts (Fig. 3a and S19), consistent with their relatively stronger PA binding energies predicted by DFT calculations (Fig. 1d). More importantly, when the same cationic group is grafted, PA uptake increases significantly with increasing crown ether content in the polymer backbone. For example, the ADC% of PTH-DMI is 68.6%, whereas that of PTC_0.3_H-DMI reaches 197.2%, nearly three times higher. This result clearly demonstrates that the introduction of crown ether units, featuring high polarity, hydrophilicity, and multiple PA interaction sites, greatly enhances the PA uptake capacity of the membranes. On the other hand, the FFV plays an important role in determining the swelling behavior of membranes during PA doping. DMI-functionalized membranes generally exhibit higher volume swelling than TEA-functionalized ones. For instance, although PTC_0.3_H-TEA shows a higher PA uptake (227.5%) than PTC_0.3_H-DMI (197.2%), its volume swelling (89.5%) is lower than that of PTC_0.3_H-DMI (99.9%). This indicates that higher FFV not only facilitates PA uptake but also helps suppress PA-induced swelling and maintain dimensional stability.^42^ Tensile stress–strain curve testing results (Fig. 3b and S20) further support this deduction: PTC_0.3_H-TEA exhibits a higher tensile strength (6.6 MPa) than PTC_0.3_H-DMI (5.9 MPa). A similar trade-off is observed with increasing DBC content. While DBC improves PA binding and uptake, its bulky and rigid structure reduces FFV, which can negatively affect dimensional stability after doping. This effect becomes particularly pronounced at high DBC content. For example, PTC_0.5_H-DMI shows excessive volume swelling (208.9%) and a low tensile strength (4.5 MPa), indicating poor mechanical robustness under practical HT-PEM operating conditions.^24,50^

**Fig. 3:**
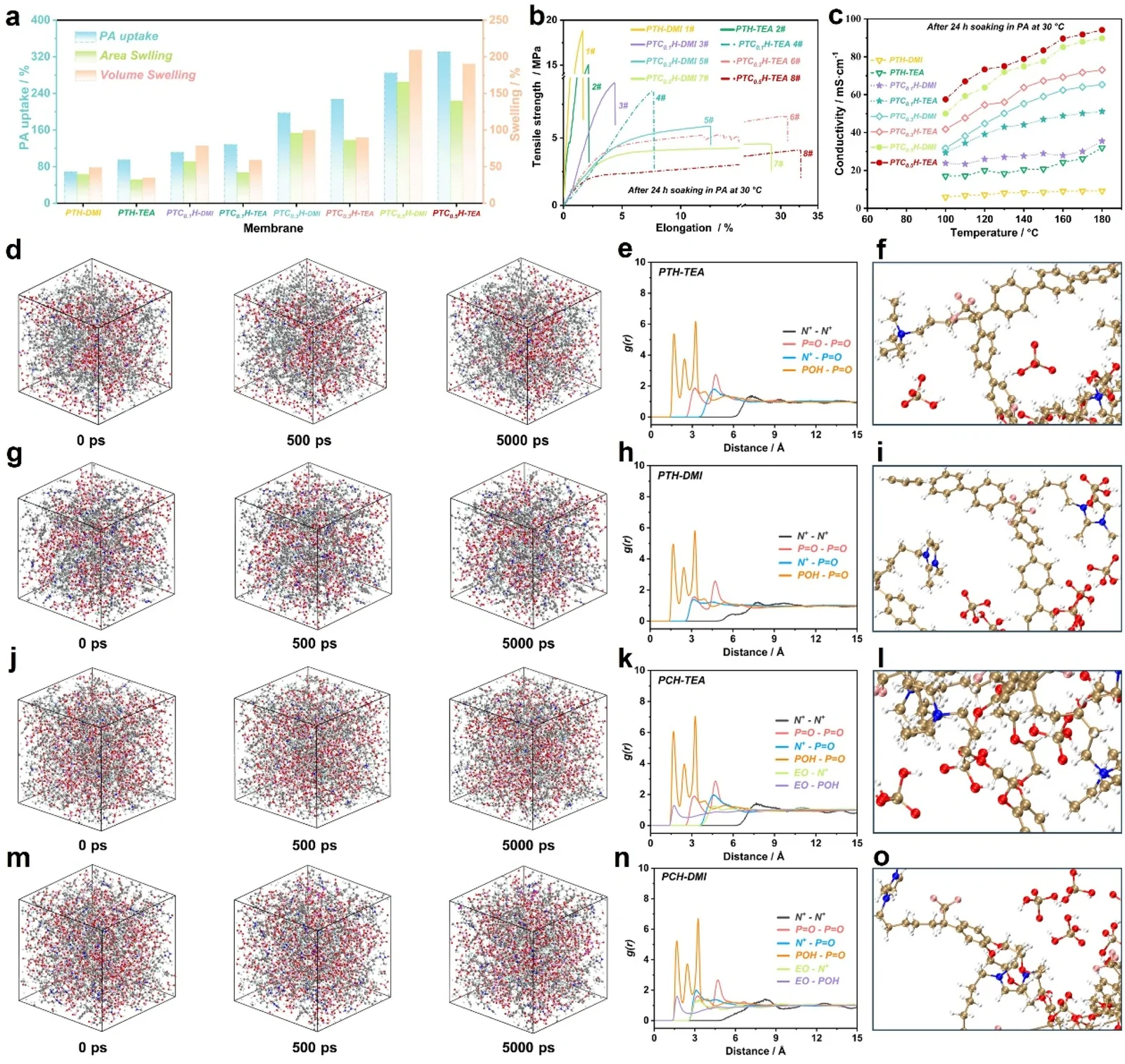
(a) PA uptake and dimensional swelling of membranes; (b) mechanical properties and (c) anhydrous proton conductivity as a function of temperature of various PA-doped membranes. Time-evolved polymer conformation, RDFs, dipole interactions and key fragment snapshots of (d–f) PTH-TEA/PA, (g–i) PTH-DMI/PA, (j–l) PCH-TEA/PA and (m–o) PCH-DMI/PA membranes.

Anhydrous proton conductivity generally increases with PA doping content. As to the pristine PTH,^12,14^ where the backbone consists solely of terphenyl units, the polymer mainly provides mechanical support, and proton transport relies entirely on the doped PA molecules (Fig. 3c and S21). As a result, PTH-TEA/PA shows significantly higher conductivity than PTH-DMI/PA due to its higher PA uptake. After introducing DBC into the backbone, additional proton transport pathways are formed through interactions between DBC and PA. At the same time, side-chain functional cation groups play a more complex role, influencing local polarity, chain mobility, and proton transport pathways. When the DBC content reaches *n* ≥ 0.1, both DMI- and TEA-functionalized membranes show a clear increase in conductivity, and the difference between them becomes much smaller. At 180 °C, PTC_0.5_H-TEA and PTC_0.5_H-DMI exhibit comparable conductivities of 94.2 and 89.8 mS cm^−1^, respectively. This improvement mainly arises from the additional conduction pathways provided by the DBC units,^40^ which complement the conventional PA-mediated mechanism.

To better understand the proton conducting behavior of various membranes, MD simulations were performed (Fig. 3d–o). Crucially, the convergence in proton conductivity between DMI- and TEA-functionalized PTC_*n*_H membranes does not originate from similar PA binding strengths, but rather from distinct polymer chain dynamics, as revealed by MD simulations. Analysis of 5000 ps MD trajectories and radial distribution functions (RDFs) of key atomic groups provides important mechanistic insights.^51,52^ For PTH-DMI, the N^+^–N^+^ RDF exhibits a pronounced oscillatory decay with a characteristic peak–valley pattern, indicating long-range lamellar ordering. This ordering arises from cooperative π–π interactions between the aromatic TP backbone and the side-chain imidazolium groups. In contrast, PTH-TEA shows only a single broad N^+^–N^+^ RDF peak, reflecting a more disordered structure dominated by the high conformational entropy of flexible alkylammonium chains and the absence of directional interactions with the backbone. For DBC-containing systems, the structural features change significantly. The N^+^–N^+^ RDF of PCH-DMI displays a single broad and gradually increasing peak, suggesting that the system is governed by multiple weak and competing non-covalent interactions. The bulky crown ether macrocycle introduces spatial separation that suppresses ion aggregation, while dipole–ion interactions between the ether oxygen atoms and the DMI^+^ cation play a dominant role. These interactions promote the formation of dynamic, localized ion-crown ether complexes, resulting in a structure with strong short-range correlations but no long-range periodicity.

Further insight is obtained from the RDF between the oxygen atoms of PA and the cationic nitrogen (N^+^) (Fig. 3e and h). In TEA-functionalized systems, the longer ethyl chains increase the average distance between the cation center and PA, leading to a shift of the N^+^–P=O RDF peak to larger distances. MD snapshots (Fig. 3f and i) confirm that TEA side chains adopt extended conformations, positioning the cation farther from the backbone, whereas DMI side chains remain closer to the aromatic backbone. This difference in spatial arrangement explains the observed variation in FFV and suggests π–π stacking between the imidazolium ring and the TP backbone in PTH-DMI. Moreover, the more compact and ordered PA adsorption network in PTH-DMI facilitates efficient proton hopping, contributing to its high conductivity.

When DBC is introduced into the backbone, the system undergoes a fundamental change in dynamic behavior. As shown in Fig. 3l, TEA side chains tend to orient toward the crown ether cavity due to favorable ion–dipole interactions. As a result, proton-conducting pathways associated with PA molecules adsorbed on DBC are no longer isolated but become interconnected through the crown ether units. This molecular-level connectivity enhances the overall transport network and highlights the advantage of the PCH architecture. In PCH-DMI, the interaction between side-chain imidazolium and the crown ether is even stronger (Fig. 3o). The imidazolium ring partially inserts into the crown ether cavity, leading to a significant reduction in the N^+^–O(crown) distance, with the first coordination peak shifting from 4.63 Å in PCH-TEA to 3.29 Å in PCH-DMI. This closer interaction indicates stronger dipole coupling and enhanced charge transfer,^51^ which together promote the formation of a more continuous and stable proton transport network. Overall, these results show that although DMI- and TEA-functionalized systems differ in their local interactions and chain dynamics, the presence of crown ether units enables both systems to achieve similarly high proton conductivity. This highlights the key role of backbone, such as side chain synergy and non-covalent interactions, in determining the performance of HT-PEMs.

To experimentally verify the proposed synergistic non-covalent interactions between DBC, cationic groups, and PA, the structural and electronic characteristics of DMI- and TEA-functionalized membranes before and after DBC incorporation and PA doping were investigated by ^1^H NMR, XPS, and FTIR spectroscopy (Fig. 2b, 4 and S22–S24). The DMI-functionalized membranes were compared with TEA-based analogues to distinguish directional aromatic interactions from general ionic interactions. ^1^H NMR spectroscopy (Fig. 2b) revealed distinct spatial interactions between DBC and the imidazolium group. After DBC incorporation, the C4–H/C5–H protons (H_e_) of the imidazolium ring exhibited downfield shifts from 7.504 ppm to 7.626 ppm (Δ*δ* = +0.122 ppm), whereas the C2–CH_3_ (H_f_) protons showed an upfield shift from 2.684 ppm to 2.556 ppm (Δ*δ* = −0.129 ppm). Such opposite chemical shift changes indicate that different regions of the imidazolium ring experience distinct shielding environments, suggesting directional recognition between the imidazolium moiety and the DBC framework. The upfield shift of C2–CH_3_ is attributed to the shielding effect from the aromatic ring current of DBC, while the downfield shift of C4–H/C5–H is associated with C–H⋯O hydrogen bonding with crown ether oxygen atoms. In contrast, TEA-functionalized membranes displayed only minor and uniform chemical shift changes after DBC incorporation (N–CH_2_– (H_c_): 3.163 to 3.191 ppm; CH_3_ (H_d_): 1.107 to 1.135 ppm; Δ*δ* < 0.03 ppm), indicating the negligible π-conjugated interactions and the dominance of weaker ion–dipole interactions.

**Fig. 4:**
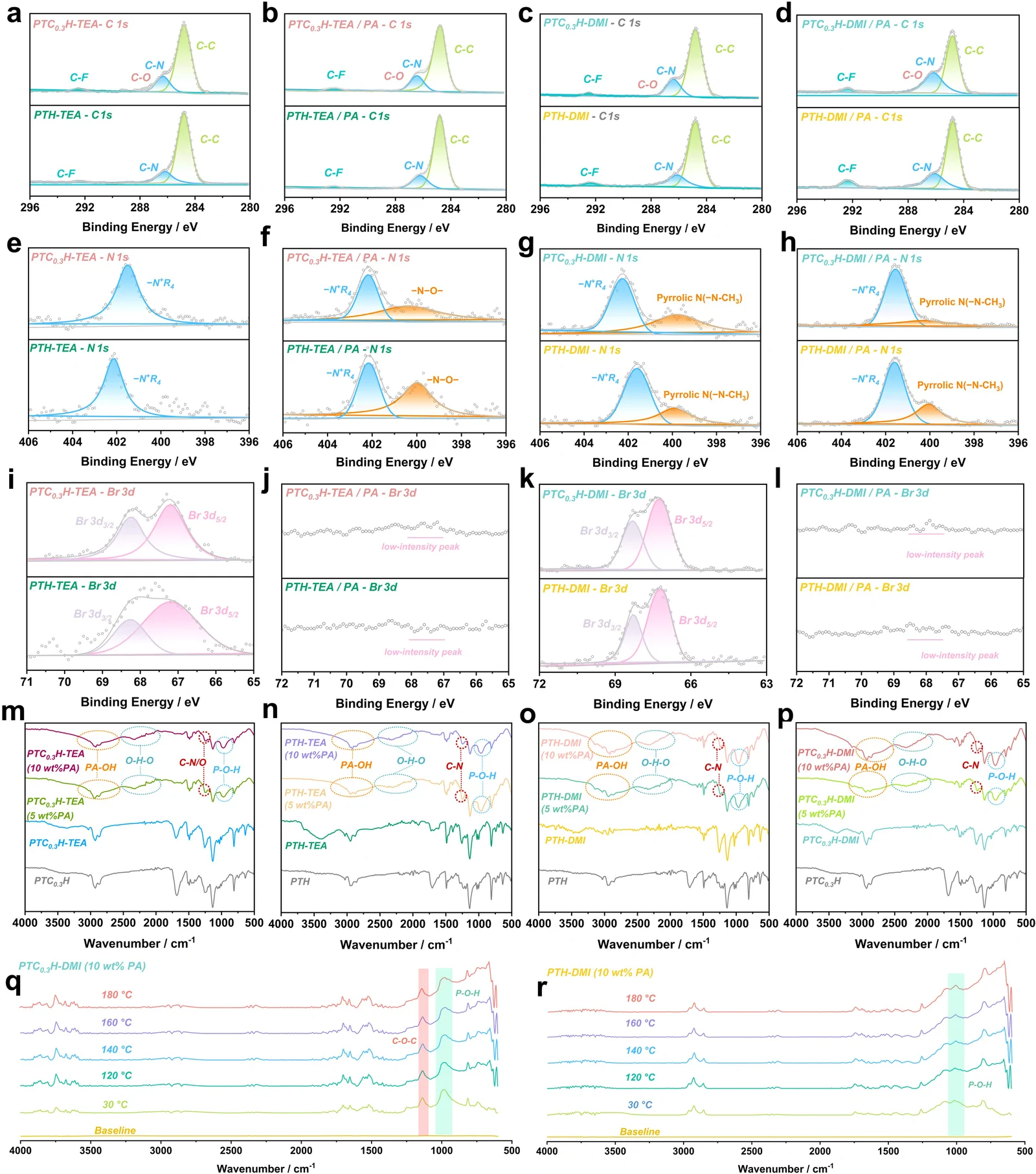
(a–d) XPS-C 1s, (e–h) XPS-N 1s, (i–l) XPS-Br 3d, (m–p) FT-IR spectra of pure PTH-TEA, PTHC_0.3_-TEA, PTH-DMI and PTC_0.3_H-DMI membranes, and above membranes after doping PA in 10% or 5% PA solutions at 30 °C. (q and r) *In situ* IR spectra of PA doped PTC_0.3_H-DMI and PTH-DMI membranes as a function of temperature.

As depicted in Fig. 4a–l, the electronic interactions between DBC and cationic groups were further examined by XPS analysis. The DMI-based membrane exhibited opposite variations in the binding energies of different nitrogen environments after DBC incorporation, demonstrating site-selective electronic modulation caused by specific DBC-imidazolium interactions. For example, for PTH-DMI, the binding energies of N1^+^ (quaternary ammonium center linked to the side chain) and N3 (tertiary nitrogen bearing only a methyl substituent) were 401.60 eV and 399.90 eV, respectively (ΔBE = 1.70 eV). For PTC_0.3_H-DMI, N1^+^ shifted to higher binding energy at 402.27 eV (ΔBE = +0.67 eV), whereas N3 shifted to lower binding energy at 399.77 eV (ΔBE = −0.13 eV), increasing the energy separation to 2.50 eV. By comparison, the TEA-based membrane showed only a uniform N 1s shift (402.14 to 401.49 eV, ΔBE = −0.65 eV), consistent with nondirectional electrostatic interactions. After PA doping, the disappearance of the Br 3d signal confirmed the exchange of Br^−^ by H_2_PO_4_^−^, while the N1^+^ binding energy in PTC_0.3_H-DMI decreased significantly from 402.27 eV to 401.55 eV (ΔBE = −0.72 eV), indicating the strong interaction between cationic groups and PA species.

FTIR analysis (Fig. 4m–p) further confirmed the DBC-induced regulation of molecular interactions. The C=N stretching vibration (imidazole ring backbone) of the DMI-based membrane shifted from 1585.38 cm^−1^ to a higher wavenumber of 1588.60 cm^−1^(Δ*ν* = +3.22 cm^−1^) after DBC incorporation, indicating enhanced conformational restriction of the imidazolium ring. Following PA doping, the smaller C–N vibration shift observed for PTC_0.3_H-DMI (−18.82 cm^−1^) compared with PTH-DMI (−19.28 cm^−1^) demonstrates that DBC effectively stabilizes the cationic structure and mitigates PA-induced electronic perturbation. However, in the TEA systems, no spectral change occurred upon DBC incorporation, the C–N peaks remaining identical at 1257.04 cm^−1^ for both PTH-TEA and PTC_0.3_H-TEA. Before PA doping, the non-directional ion–dipole interaction between DBC and TEA is already conformationally averaged due to the high flexibility of the TEA side chain, and is further masked by the electrostatic shielding of Br^−^ counterions. Under these conditions, FTIR spectra, probing the instantaneous vibrational energy levels of chemical bonds, cannot resolve the subtle spectral shifts arising from a dynamic ensemble of weakly interacting conformations. Upon PA doping, however, the C–N redshift of PTC_0.3_H-TEA (−9.18 cm^−1^) exceeded that of PTH-TEA (−7.25 cm^−1^), reflecting the liberation of the DBC-TEA ion–dipole interaction upon Br^−^ removal. Together, these spectroscopic results provide direct experimental evidence that DBC interacts with imidazolium groups through cooperative π–π stacking, C–H⋯O hydrogen bonding, and ion–dipole interactions, thereby regulating PA distribution and reinforcing the proton transport network.

To further evaluate the stability of the hydrogen-bonding network and PA-binding interactions, *in situ* variable-temperature FTIR spectroscopy was performed for PTH-DMI/PA and PTC_0.3_H-DMI/PA (Fig. 4q and r). In the fingerprint region, PTH-DMI/PA exhibits relatively broad and overlapped absorption features, whereas PTC_0.3_H-DMI/PA displays significantly enhanced and well-defined characteristic peaks at approximately 1130 cm^−1^ and 980 cm^−1^, assigned to the C–O–C stretching vibration of the crown ether units and the P–O–H vibration of phosphoric acid, respectively. The enhanced spectral definition might be attributed to the increased local polarization and structural ordering induced by the electron-rich DBC units, which could create a more stable coordination environment for PA molecules. Notably, these characteristic peaks remain almost unchanged during continuous heating from 30 to 180 °C, with no obvious peak displacement, broadening, or intensity attenuation, indicating that the PA-binding configuration remains stable under elevated-temperature conditions. It should be noted that the broad hydrogen-bonding related bands located around 3300–3400 cm^−1^ are highly sensitive to background subtraction and water evaporation effects during *in situ* high-temperature measurements. Therefore, these bands were not selected as the primary indicators for evaluating the stability of the hydrogen-bonding network. Collectively, these results demonstrate that the introduction of DBC units establishes a robust and thermally stable hydrogen-bonding network, which contributes to effective PA retention and efficient proton transport under high-temperature anhydrous conditions.

### 2.4 Thermal, chemical and PA stabilities

The overall stability of HT-PEMs is a key factor that determines their long-term operation. Thermogravimetric analysis (TGA) was used to evaluate the thermal stability of the membranes (Fig. 5a and S25). The initial weight loss below 250 °C is mainly due to the removal of residual solvent and adsorbed water.^29^ In the range of 250–500 °C, weight loss is primarily associated with the decomposition of alkyl side chains and DBC units within the polymer backbone.^32,34,40^ At higher temperatures, gradual mass loss corresponds to the degradation of the aromatic framework.^24^ Moreover, functionalization of cationic groups generally reduces thermal stability of copolymers, as evidenced by the additional weight loss observed for PTC_0.3_H-DMI and PTC_0.3_H-TEA in the intermediate temperature range (250–500 °C).^32^ Differential thermogravimetric (DTG) analysis (Fig. 5b and S26) shows that the main decomposition temperatures are approximately 400 °C for PTC_0.3_H-TEA, 420 °C for PTC_0.3_H-NMI, and 450 °C for PTC_0.3_H-DMI. These results indicate that imidazolium-functionalized membranes have higher thermal stability than quaternary ammonium-based systems. This improvement can be attributed to the π-conjugated imidazolium, which allows charge delocalization and increases resonance stability. Furthermore, PTC_0.3_H-DMI shows better thermal stability than PTC_0.3_H-NMI. The methyl group at the C2 position in DMI suppresses the low-energy proton transfer pathway involving the C2–H bond, forcing degradation to proceed through higher-energy bond cleavage. At the same time, the electron-donating effect of the methyl groups increases electron density around the imidazolium ring, strengthening the C–N bond and stabilizing the cationic center.

**Fig. 5:**
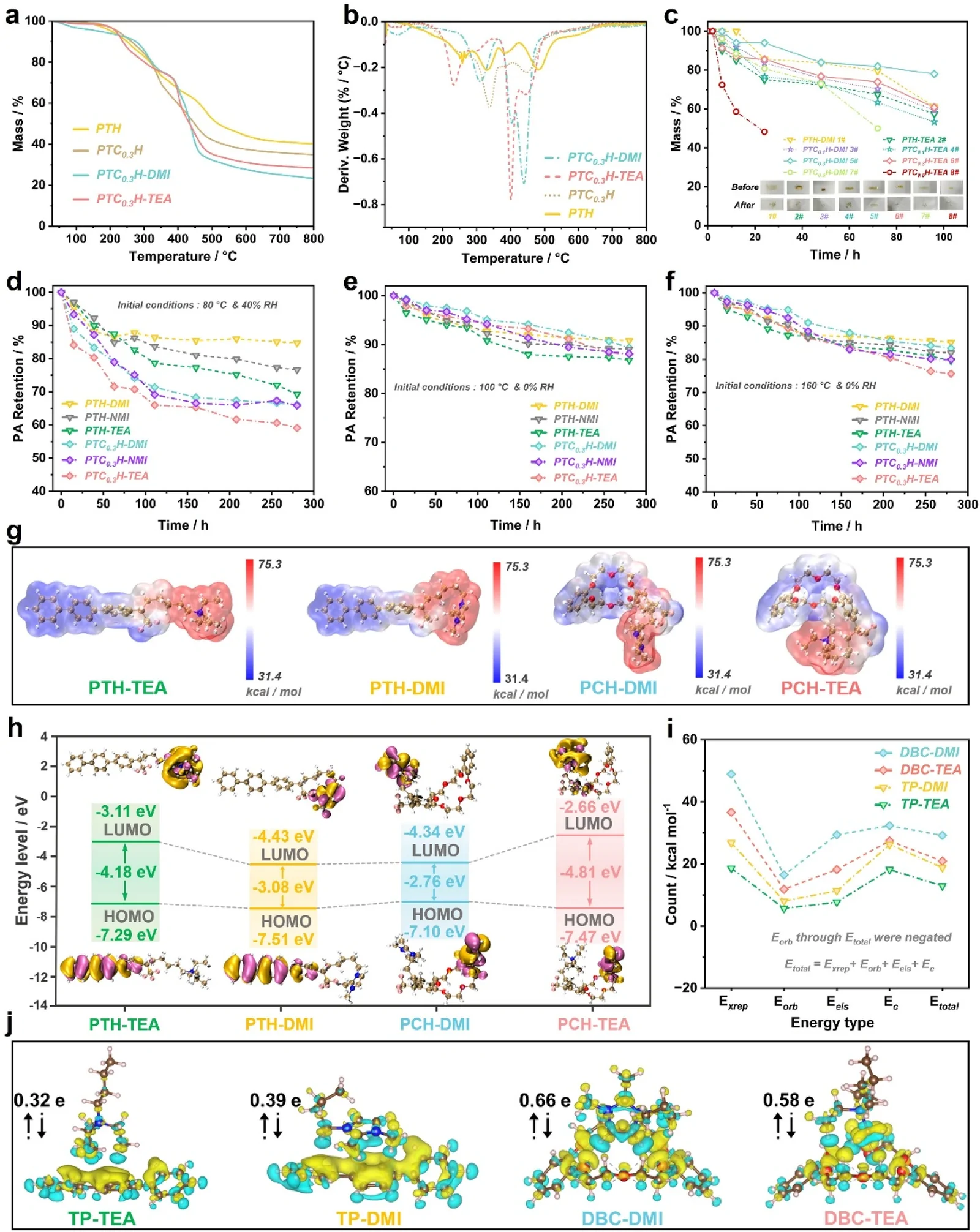
(a) TGA curves; (b) DTG curves and (c) Fenton test (in 3% H_2_O_2_ and 3 ppm Fe^2+^ at 80 °C) results including digital photos of membranes before and after Fenton test. PA retention under various conditions: (d) 80 °C/40% RH, (e) 100 °C/0% RH and (f) 160 °C/0% RH; (g) molecular ESP and (h) orbital energies of PTH-TEA, PTH-DMI, PCH-DMI and PCH-TEA; (i) EDA and (j) charge density difference analyses for DBC/TP-DMI/TEA interactions.

Chemical stability was evaluated using Fenton's test, which simulates oxidative degradation by hydroxyl radicals (Fig. 5c and S27).^5,53^ After 96 h of Fenton treatment, all DBC-containing copolymer membranes exhibited noticeable changes in color intensity, which are attributed to oxidative degradation of the crown ether units and the resulting modification of the local conjugation and intermolecular interaction environments. PTH-TEA shows significant degradation with a mass loss of 42.5%, whereas PTH-DMI exhibits a lower mass loss (39.1%) and better structural integrity. This improved stability can be attributed to the π-conjugated imidazolium ring and the protective effect of the C2-methyl substituent, which together enhance resistance to oxidative attack. The introduction of DBC further improves oxidative stability.^43^ Among all samples, PTC_0.3_H-DMI shows the best performance, with nearly 80% mass retention and well-preserved membrane morphology. It should be noted that both the ether linkages within DBC units and the side-chain quaternary ammonium groups are intrinsically susceptible to oxidative degradation under strongly oxidative conditions. Compared with the previously reported fully aromatic DBC-containing pyridine membrane (*i.e.*, P(TP_91%_-co-CE_9%_)) containing only 9% DBC units,^40^ the PTC_0.3_H-DMI membrane exhibits slightly inferior oxidative stability, as demonstrated by the Fenton test results in Fig. S27a. However, when the DBC content was further increased to 27%, the P(TP_73%_-co-CE_27%_) membrane displayed a more pronounced deterioration in chemical stability than PTC_0.3_H-DMI. These results emphasize the critical role of the non-covalent interaction network formed between the crown ether backbone and flexible cationic side chains, which effectively regulates the local molecular environment, protects vulnerable structural units, and maintains membrane integrity during oxidative degradation.

MD simulations and electronic structure analysis provide insight into this enhancement. Charge density difference and energy decomposition analysis (Fig. 5g–j) reveal stronger interactions between DBC and DMI than between DBC and TEA. For example, the DBC-DMI fragment gains 0.66 *e*^−^ from neighboring DBC units, which is higher than the 0.58 *e*^−^ observed for the DBC-TEA system. In addition, DBC-DMI system shows a higher electrostatic interaction energy (*E*_els_). Although its exchange repulsion energy (*E*_rep_) is slightly higher than that of DBC-TEA, the overall interaction energy remains strong (Fig. 5i). These results indicate enhanced dipole coupling in the DBC-DMI system, which stabilizes the polymer structure and improves resistance to radical attack. However, the stabilizing effect of DBC depends strongly on its content.^40,42^ At high DBC content, the crown ether segments become more vulnerable to oxidative degradation. For example, PTC_0.5_H-TEA retains only 48.3% of its mass and completely loses mechanical integrity within 24 h. Similarly, PTC_0.5_H-DMI shows initial stability but undergoes rapid degradation after 48 h. These results indicate that excessive incorporation of DBC can reduce overall chemical stability, highlighting the need to optimize its content for balanced performance.

Proton conduction in HT-PEMs relies on a stable hydrogen-bond network formed by PA molecules. Therefore, PA retention is a critical factor for long-term performance.^12,14,37^ PA leaching behavior was evaluated under different temperature and humidity conditions (Fig. 5d–f). Imidazolium groups (NMI^+^ and DMI^+^) interact strongly with phosphate species through acid–base and electrostatic interactions, and methyl substitution further enhances this interaction by increasing alkalinity and charge delocalization. As a result, DMI-functionalized membranes show better PA retention than NMI-based systems. Environmental conditions strongly affect PA stability.^53^ Under high-humidification and low-temperature conditions, water uptake can accelerate PA loss due to dilution and diffusion. However, these conditions are less relevant to actual fuel cell operation, which typically involves high temperatures and low humidity. Under anhydrous and high-temperature conditions (160 °C), PA loss becomes more severe due to increased chain mobility and disruption of hydrogen-bond networks.^29,54^ In this case, the crown ether units play an important role. Membranes containing DBC, such as PTC_0.3_H-DMI, show excellent PA retention even after long-term operation. After nearly 300 h, PTC_0.3_H-DMI maintains PA retention comparable to PTH-DMI despite its higher ADC%. Moreover, the PTC_0.3_H-DMI membrane exhibits improved PA retention compared with the previously reported P(TP_91%_-co-CE_9%_) membrane under elevated temperature anhydrous condition (*i.e.*, 160 °C and 0% RH), while maintaining comparable PA retention under moderate humidity conditions (80 °C and 40% RH), as shown in Fig. S28. These results demonstrate that the stronger ionic interaction between imidazolium groups and phosphate species, together with the additional stabilization from DBC-cation interactions, effectively contribute to enhanced PA retention of PTC_0.3_H-DMI, particularly under high-temperature operating conditions. Overall, these results demonstrate that the combination of C2-methyl substituent imidazolium-functionalized side chains and crown ether-containing backbones provides a balanced improvement in thermal stability, chemical resistance, and PA retention. This synergistic design is essential for achieving high-performance HT-PEMs under practical operating conditions.

### 2.5 Fuel cell performance

A comprehensive comparison of membrane properties is summarized in the radar chart shown in Fig. 6a. Based on the complementary advantages identified for PTC_0.3_H-TEA and PTC_0.3_H-DMI, both membranes were assembled into membrane electrode assemblies (MEAs) using Pt/C catalysts and evaluated under unhumidified H_2_/O_2_ conditions without backpressure. Polarization and power density curves were recorded at temperatures above 120 °C with 20 °C intervals.^22,26^ As shown in Fig. 6b, the cell with PTC_0.3_H-TEA exhibits high proton conductivity, but its relatively low open-circuit voltage (OCV, approximately 0.81 V) limits the overall power output. At 180 °C, it achieves a peak power density of 785 mW cm^−2^, corresponding to a 157% increase compared with its performance at 120 °C. In contrast, PTC_0.3_H-DMI delivers a much higher peak power density of 1152 mW cm^−2^ at 180 °C (Fig. 6c), although the relative increase over the same temperature range is smaller (122%). This difference can be understood from their distinct chemical structures. The PTC_0.3_H-TEA membrane forms a highly continuous hydrogen-bond network, which facilitates efficient proton transport through the Grotthuss mechanism and leads to strong temperature dependence.^34,44,55^ However, this structure also results in weaker gas barrier properties, contributing to its lower OCV. In comparison, the PTC_0.3_H-DMI membrane shows a more optimized free-volume distribution and suitable hydrogen-bond continuity, which significantly improves gas barrier performance, resulting in a higher OCV of about 0.88 V. Hydrogen crossover measurements using linear sweep voltammetry (LSV) confirm that PTC_0.3_H-DMI/PA exhibits lower crossover current densities than PTC_0.3_H-TEA/PA over the investigated temperature range (Fig. S29). Specifically, at 160 °C, the hydrogen crossover current density of PTC_0.3_H-DMI/PA is 2.21 mA cm^−2^, which is approximately 11% lower than that of PTC_0.3_H-TEA/PA (2.47 mA cm^−2^). Similar trends are observed at 120 °C (1.78 *vs.* 1.93 mA cm^−2^) and 140 °C (1.88 *vs.* 2.08 mA cm^−2^). As a result, PTC_0.3_H-DMI achieves superior overall performance and maintains its advantage across the entire high-temperature range.

**Fig. 6:**
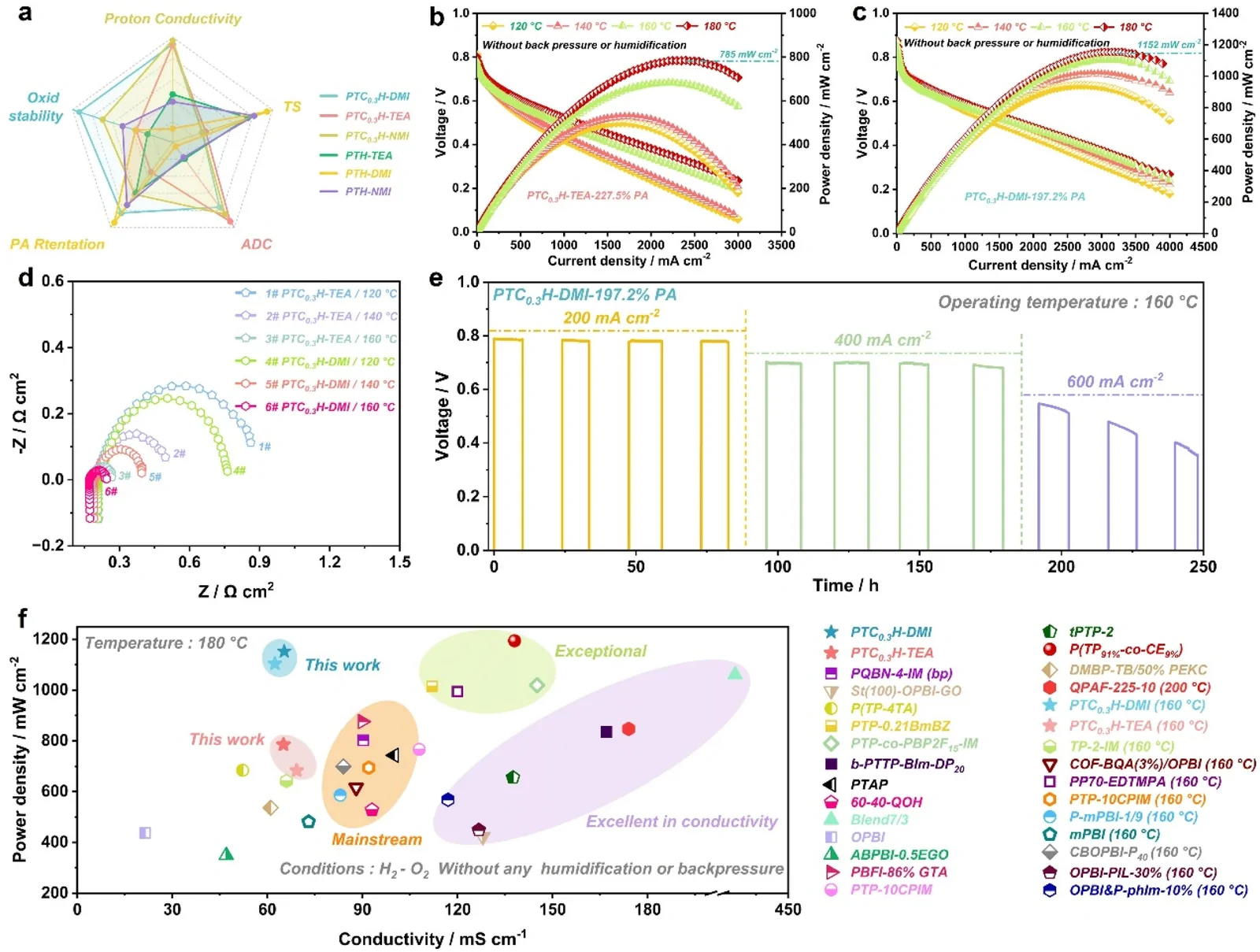
(a) Comprehensive performance comparison of HT-PEMs; (b and c) single cell performance and (d) EIS curves of the single cells with PTC_0.3_H-TEA/PA and PTC_0.3_H-DMI/PA membranes under H_2_–O_2_ with no humidification and no back pressure; (e) start-stop cycle test of the cell with PTC_0.3_H-DMI/PA at 160 °C under different current densities; (f) comparative performance of HT-PEMs in this study and literature.

Although PTC_0.3_H-TEA/PA exhibits higher *ex situ* proton conductivity, this advantage does not translate into superior fuel cell performance. The nearly identical ohmic resistance values indicate that bulk proton transport is not the limiting factor. Instead, the lower hydrogen crossover and reduced charge-transfer resistance of PTC_0.3_H-DMI/PA demonstrate the importance of controlling membrane microstructure and electrode interface stability. As demonstrated by electrochemical impedance spectra (EIS) in Fig. 6d, the ohmic resistance (*R*_ohm_) of PTC_0.3_H-DMI/PA (0.17182 Ω cm^2^) is nearly identical to that of PTC_0.3_H-TEA/PA (0.17543 Ω cm^2^, difference 0.00361 Ω cm^2^), whereas the charge-transfer resistance (*R*_ct_) of PTC_0.3_H-DMI/PA (0.24530 Ω cm^2^) is significantly lower than that of PTC_0.3_H-TEA/PA (0.26610 Ω cm^2^, difference 0.02080 Ω cm^2^, nearly six times the *R*_ohm_ difference). Therefore, these results highlight that effective HT-PEM design requires simultaneous optimization of proton conduction, gas separation, and PA retention rather than conductivity enhancement alone. To further evaluate practical durability, the single cell based on PTC_0.3_H-DMI/197.2% PA was operated under stepped current densities combined with start-stop cycling at 160 °C as depicted in Fig. 6e. The cell maintained stable voltage output under 200 and 400 mA cm^−2^ operation for prolonged periods. When the current density was increased to 600 mA cm^−2^, accelerated degradation occurred due to enhanced water generation at the cathode and increased PA loss. Nevertheless, compared with the previously reported P(TP_91%_-co-CE_9%_)/PA membrane, which experienced rapid voltage decay within 5 h under 400 mA cm^−2^ at 160 °C (Fig. S30), the PTC_0.3_H-DMI based MEA demonstrated significantly improved operational stability. These results confirm that the non-covalent interaction network plays a critical role in stabilizing both the polymer matrix and the acid-mediated proton transport pathways.

Fig. 6f compares the performance of the membranes in this work with representative HT-PEMs reported in the literature. It should be noted that such comparisons are qualitative due to differences in testing conditions, including gas flow rates, electrode structures, and cell configurations.^22,56^ Most reported systems fall within a typical performance range, while only a few advanced materials reach very high power densities close to 1200 mW cm^−2^. Notably, the PTC_0.3_H-DMI membrane achieves excellent power output despite having only moderate conductivity. This result highlights the importance of efficient PA management and well-designed microstructure, rather than relying solely on high conductivity. The strong interaction between PA and the imidazolium groups, combined with the well-organized transport pathways provided by the crown ether units, enables efficient proton transport under real operating conditions. Overall, these results suggest that future HT-PEM design should move beyond simply increasing PA uptake. Instead, greater emphasis should be placed on controlling intermolecular interactions and microstructure to achieve a balanced improvement in conductivity, stability, and fuel cell performance.

## 3 Conclusions

In summary, we developed a series of high-performance HT-PEMs by integrating dibenzo-18-crown-6 (DBC) units with abundant lone pair electrons into a long-side-chain poly(arylene alkylene) framework, followed by functionalization with different cationic groups. This work demonstrates a design strategy that couples backbone engineering with side-chain functionalization to regulate intermolecular interactions and membrane microstructure. The incorporation of DBC introduces strong dipole interactions and additional PA binding sites, which enhance hydrophilicity, promote well-defined phase separation, and improve PA uptake. At the same time, grafted imidazolium groups, particularly with C2-methyl substitution, provide high chemical stability and strong PA retention through charge delocalization and electronic stabilization. Molecular simulations reveal that the synergy between crown ether units and imidazolium side chains governs chain dynamics and enables the formation of efficient and continuous proton transport pathways. As a result, the optimized PTC_0.3_H-DMI membrane exhibits excellent stability and fuel cell performance, retaining 83.3% of PA after 280 h at 160 °C under anhydrous conditions and delivering a peak power density of 1152 mW cm^−2^ at 180 °C without humidification or backpressure. Meanwhile the cell also maintained stable voltage during long-term operation under 200 and 400 mA cm^−2^ at 160 °C. Overall, this study highlights the critical role of non-covalent interactions in controlling proton transport, chemical stability, and acid retention. It provides a practical molecular design approach for next-generation HT-PEMs, shifting the focus from simply increasing acid uptake to engineering well-defined intermolecular interactions and microstructures for balanced and durable performance.

## Author contributions

The manuscript was written through contributions from all authors.

## Conflicts of interest

There are no conflicts to declare.

## Supplementary Material

SC-017-D6SC04018G-s001

## Data Availability

The data supporting this article have been included as part of the supplementary information (SI). Supplementary information: descriptions related to polymer synthesis, theoretical calculation methods, membrane preparation procedures, single-cell testing protocols, and additional figures and tables supporting this article. See DOI: https://doi.org/10.1039/d6sc04018g.
