# Supplementary material for: Synergistic backbone-side chain design via noncovalent interactions for advanced high-temperature proton exchange membranes
Source: Chem Sci. 2026 Aug 11;17(39):18748–62. doi: 10.1039/d6sc04018g (PMC13482904; doi:10.1039/d6sc04018g)
Supplement: Supplementary file 1 [file SC-017-D6SC04018G-s001.pdf]

Supplementary Information (SI) for Chemical Science.
This journal is © The Royal Society of Chemistry 2026

Supporting Information

Synergistic Backbone-Side Chain Design via Noncovalent Interactions for Advanced High-Temperature Proton Exchange Membranes

Qian Wang[a,1], Wenzhe Zhao[a,1], Zhejing Zhang[a,1],Yang Wu[b] and Jingshuai Yang[a,*]

a. Department of Chemistry, College of Sciences, Northeastern University, Shenyang 110819, China

b. College of Chemistry, Liaoning University, Shenyang 110036, Liaoning, China

1. These authors contributed equally to this work.

* Corresponding author: yjs@mail.neu.edu.cn (J. Yang)

Table of Contents

Material 2
Synthesis of PTH and PTCnH 2
Preparation of functionalized polymers 3
Preparation of membranes 3
Computational methods 4
Fuel Cell Testing 5
Supplementary figures 6
Supplementary tables 20
References 23

Material

Dibenzo-18-crown-6 (DBC), p-terphenyl (TP), 7-bromo-1,1,1-trifluoro-2-heptanone, triethylamine (TEA), N-methylimidazole (NMI), 1,2-dimethylimidazole (DMI) and trifluoromethanesulfonic acid (TFSA) were supplied by Adamas Reagent Ltd. Other chemicals, including phosphoric acid (PA), dichloromethane (DCM), sodium bicarbonate ($NaHCO_3$), N-Methylpyrrolidone (NMP), Trichloromethane, dimethyl sulfoxide (DMSO) were obtained from Sinopharm Chemical Reagent Co. Ltd. All reagents were used without further purification.

Synthesis of PTH and PTCnH

A series of PTC_nH polymers were synthesized via acid-catalyzed Friedel-Crafts alkylation by reacting p-terphenyl (0.50 g, 2.1711 mmol) with varying amounts of dibenzo-18-crown-6 and 7-bromo-1,1,1-trifluoro-2-heptanone in DCM using TFSA as the catalyst within a 150 mL two-necked round-bottom flask. Specifically, for PTH (n=0), TP (0.50 g, 2.1711 mmol) and 7-bromo-1,1,1-trifluoro-2-heptanone (0.7037 g, 2.8371 mmol) in 2.3 mL DCM were stirred under an ice bath, followed by the dropwise addition of 1 mL TFSA; after 2 hours, the resulting light purple viscous solution was poured into 500 mL of 1.0 mol L^{-1} $NaHCO_3$ to precipitate a white polymer, which was then cut, soaked in the alkaline solution for 24 h to remove residual acid, filtered, washed with deionized water until neutral, and vacuum-dried at 80 °C for 24 h before grinding and storage. For $PTC_{0.1}H$ (n=0.1), the reaction mixture included TP (0.50 g, 2.1711 mmol), DBC (0.0866 g, 0.2430 mmol), and 7-bromo-1,1,1-trifluoro-2-heptanone (0.7768 g, 3.1443 mmol) in 2.3 mL DCM with 0.5 mL TFSA, yielding a purple viscous solution after 12 h before undergoing the same workup procedure. For

$PTC_{0.3}H$ (n=0.3), TP (0.50 g, 2.1711 mmol) was reacted with DBC (0.3345 g, 0.9281 mmol) and 7-bromo-1,1,1-trifluoro-2-heptanone (0.9990 g, 4.0437 mmol) in 2.3 mL DCM with 1 mL TFSA. Finally, for $PTC_{0.5}H$ (n=0.5), the synthesis involved TP (0.50 g, 2.1711 mmol), DBC (0.7820 g, 2.1711 mmol), and 7-bromo-1,1,1-trifluoro-2-heptanone (1.3946 g, 5.6448 mmol) in 4 mL DCM with 2 mL TFSA, which produced a brown-black viscous solution after 72 h, followed by the identical purification and drying protocol described for the n=0 sample.

Preparation of functionalized polymers

Functionalized polymers bearing three distinct types of alkaline groups were prepared by grafting them onto the polymer side chains via the Menshutkin reaction. Taking the synthesis of $PTC_{0.3}H$-DMI as a representative example: 0.15 g (0.3010 mmol) of $PTC_{0.3}H$ was dissolved in 8 g of NMP in a 20 mL glass vial to form a homogeneous, transparent light-yellow solution. Simultaneously, DMI (1.5049 mmol) was dissolved in NMP to prepare a 10 wt% solution. The two solutions were then mixed and subjected to continuous magnetic stirring at 80 °C for 24 h to facilitate the grafting reaction. Upon completion, the reaction mixture was filtered and uniformly cast onto a Petri dish. The cast membrane was dried in a vacuum oven at 80 °C for 30 h and subsequently peeled off to obtain the $PTC_{0.3}H$-DMI membrane. Finally, the membrane was washed with hot deionized water under boiling conditions to remove residual solvent and excess alkaline groups, then dried and stored in a sealed container.

Preparation of membranes

All membranes were fabricated using the solution casting method. Taking the

preparation of PTC_nH membranes as a representative example: 0.15 g of PTC_nH polymer was dissolved in 8 g of NMP to form a homogeneous, transparent, light-yellow solution. The resulting solution was centrifuged and allowed to stand, followed by filtration to remove any insoluble impurities. The filtered solution was then uniformly cast onto the surface of a Petri dish using a dropper. The cast membrane was dried in a vacuum oven at 80 °C for 30 h and subsequently peeled off to obtain the pristine PTC_nH membrane. Finally, the membrane was washed with boiling deionized water to remove residual solvent from the surface, dried, and stored in a sealed container.

Computational methods

All quantum chemical calculations in this study were performed within the framework of density functional theory. Geometry optimizations and frequency calculations were carried out using the Gaussian 16 C.01 package at the B3LYP/def-TZVP level of theory, with D3(BJ) dispersion correction to accurately describe long-range dispersion interactions [1]. To ensure that the structures correspond to minima on the potential energy surface, vibrational frequency analyses were performed at the same level of theory for all optimized geometries, and no imaginary frequencies were found. To obtain accurate electronic structure information, single-point energy calculations were subsequently performed on the optimized geometries using a larger basis set, def2-TZVP. The resulting wavefunction data were used for further electronic structure analyses. Solvent effects were simulated for an aqueous environment using the SMD continuum solvation model. The non-equilibrium solvation free energy correction, combined with thermodynamic integration methods, was employed to effectively

characterize solute–solvent interactions. Electronic structure analyses, including molecular orbital distributions, electrostatic potential (ESP), condensed dual descriptor (CDD), and electron density difference (EDD), were all performed with the Multiwfn_3.8_dev program [2,3]. Visualization was achieved using VMD 1.9.3.

The polymer systems were modeled by classical molecular dynamics (MD) simulations employing the COMPASS force field [4]. For each system, fifteen chains, each consisting of five repeat units, were randomly inserted into a simulation box at 298 K. The initial configurations were relaxed by conjugate-gradient energy minimization to remove unfavorable contacts. Equilibration was subsequently conducted in the isothermal–isobaric (NPT) ensemble at 298 K and 1 atm for 1 ns, with temperature and pressure controlled by the V-rescale thermostat and the Berendsen barostat, respectively. Following equilibration, a 5 ns production run was performed under the same conditions, using a Nosé–Hoover thermostat and barostat. The equations of motion were integrated with a time step of 1 fs. Van der Waals interactions were truncated at a cutoff distance of 1.2 nm, and long-range electrostatic interactions were treated using the particle–particle particle–mesh (PPPM) method with the same cutoff. Atomic coordinates were saved every 1000 steps (1 ps) for subsequent analysis.

Fuel Cell Testing

Membrane electrode assemblies (MEAs) were fabricated via a hot-pressing method. Specifically, two gas diffusion electrodes (GDEs) with an active area of 1.5 cm^2 were hot-pressed onto both sides of the PA-doped and equilibrated membrane at

110 ℃ under a pressure of 2 MPa for 1.5 min. The resulting effective electrode area was 1 cm^2, with a platinum loading of 0.9 mg cm^{-2}. Fuel cell performance was evaluated using dry hydrogen and oxygen fed at flow rates of 120 mL min^{-1} and 60 mL min^{-1}, respectively. The tests were conducted over a temperature range of 120-180 ℃ under ambient pressure (i.e., without back pressure).

Supplementary figures

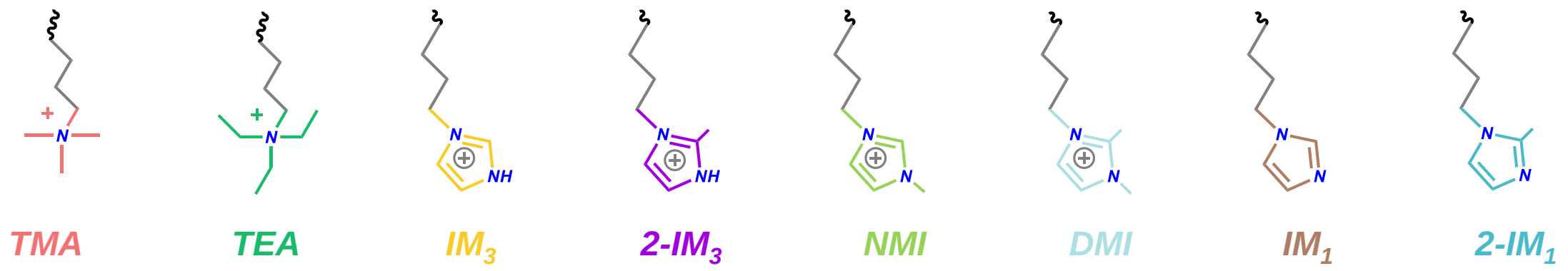

Fig. S1 The chemical structures of the relevant molecules involved.

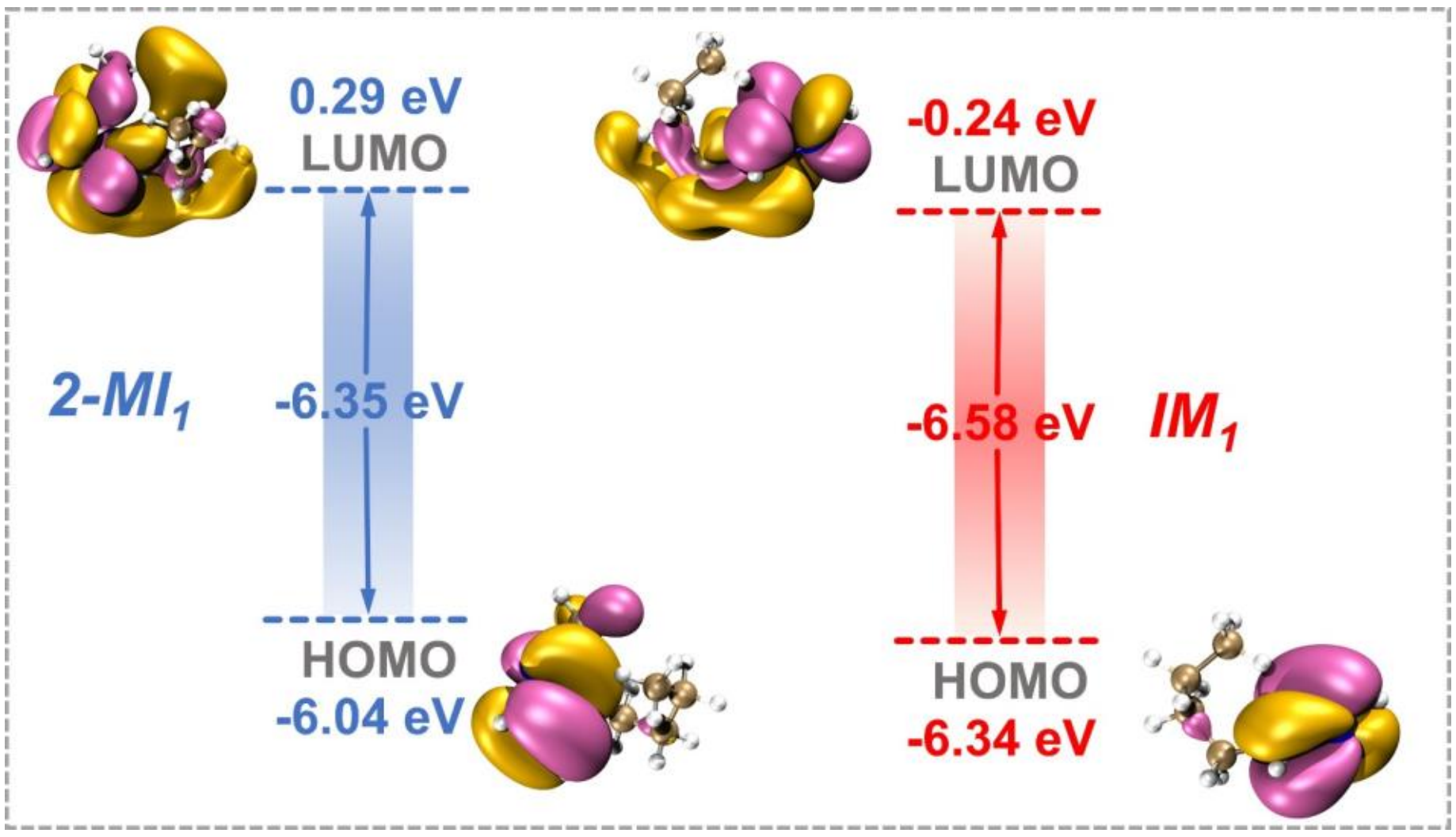

Fig. S2 Energy level orbital information of 2-MI_1 and IM_1.

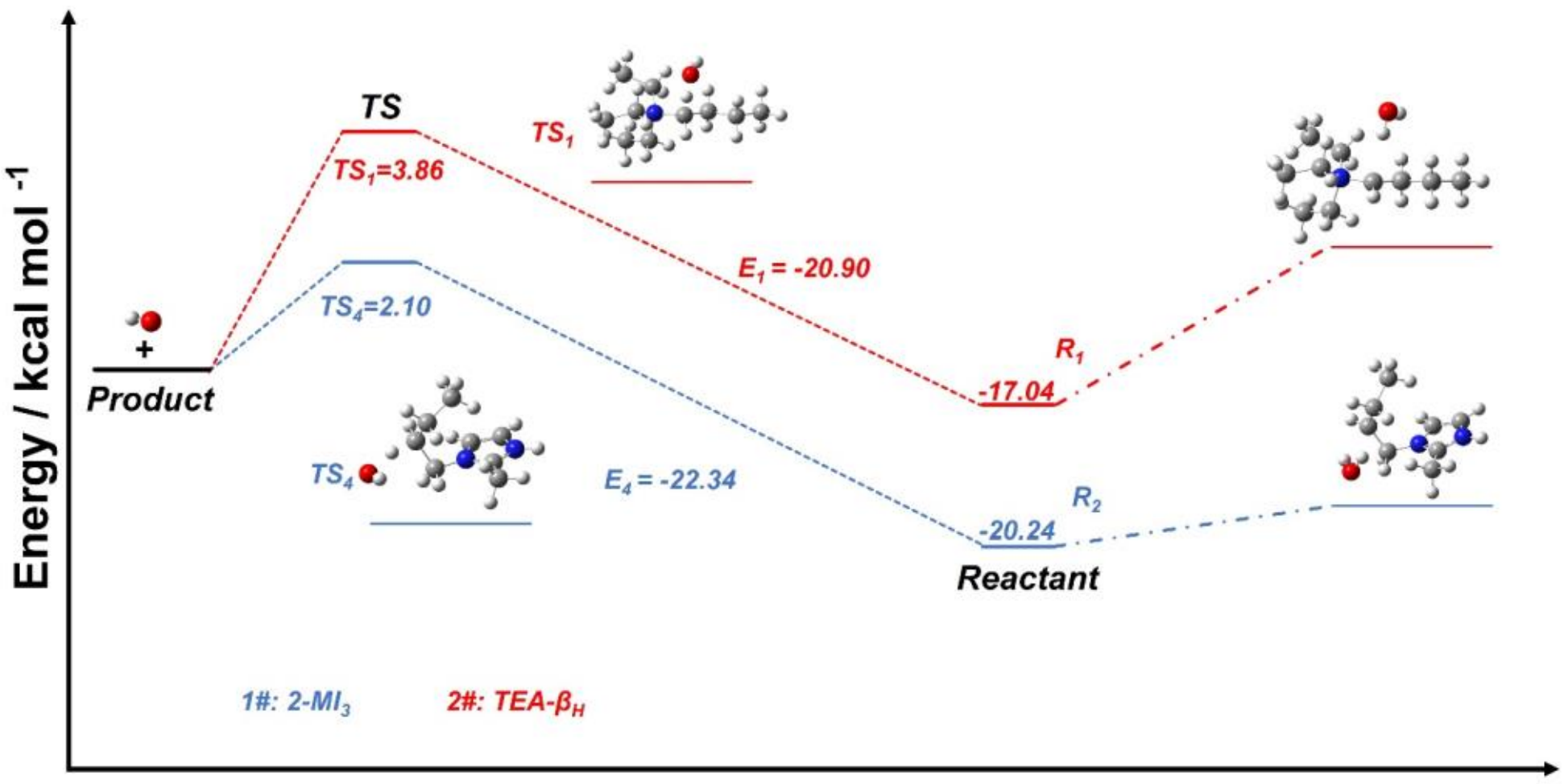

Fig. S3 Transition states for radical attack of 2-MI$_3$ and TEA-β_H.

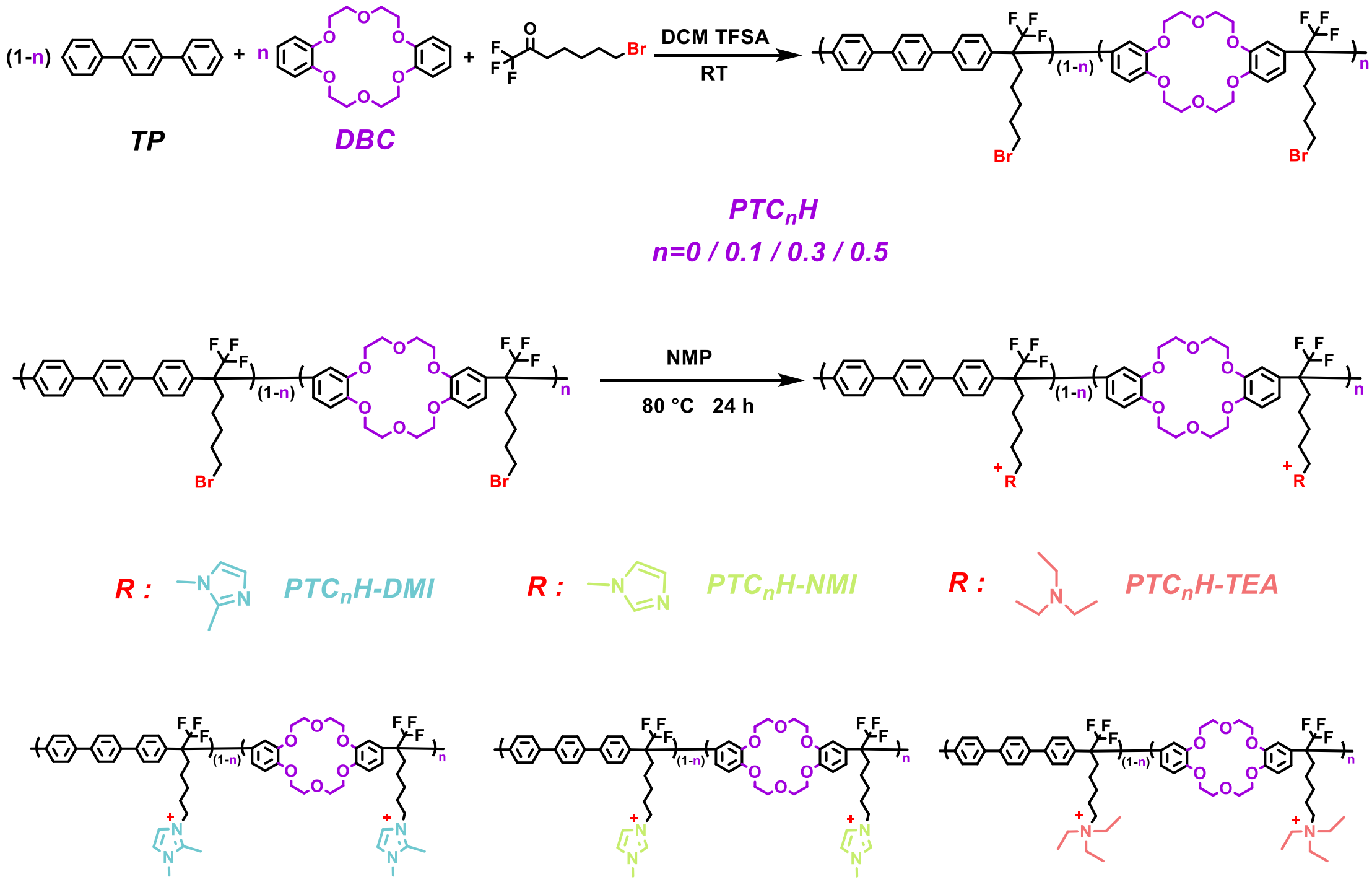

Fig. S4 Polymer synthesis of series PTC$_n$H copolymers.

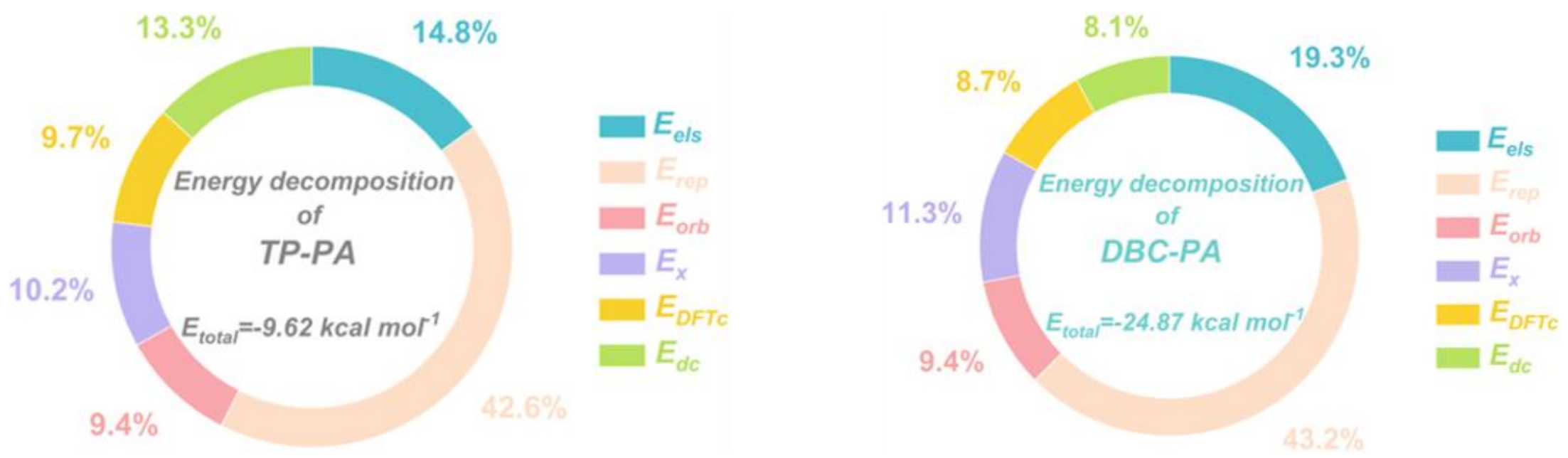

Fig. S5 Energy decomposition analysis of TP/DBC-PA interactions.

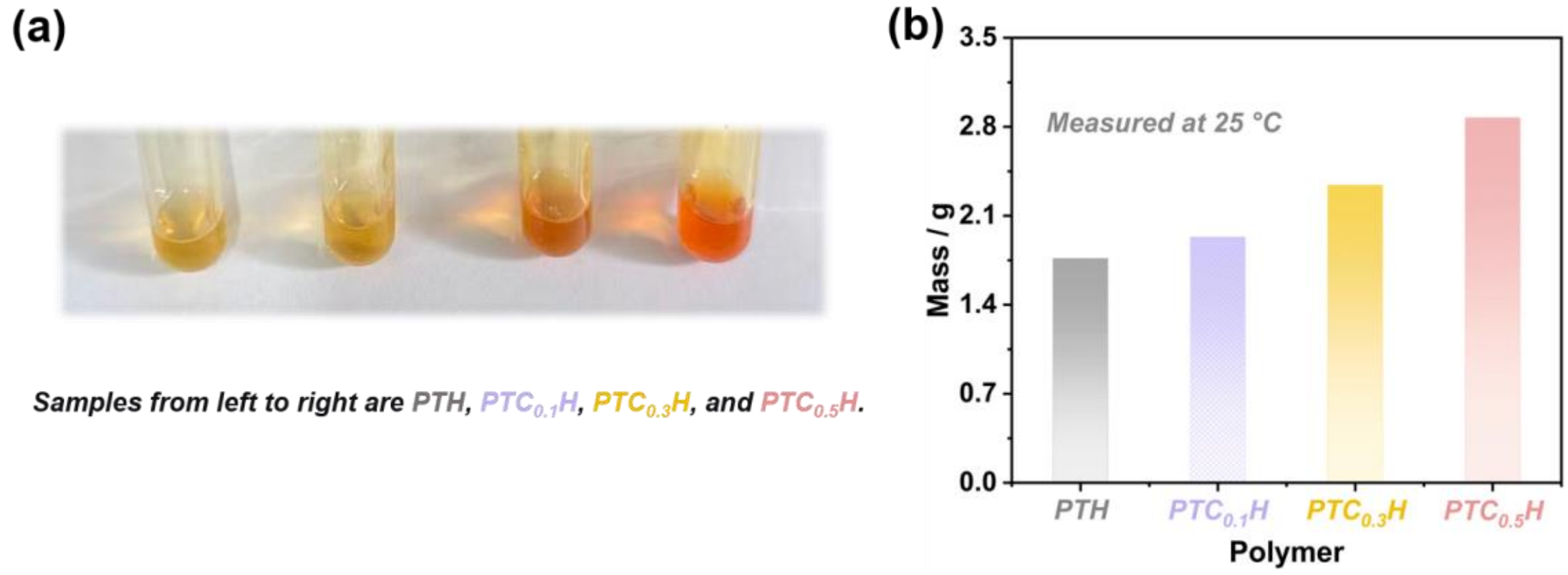

Fig. S6 The maximum soluble amount of PTH and PTC$_n$H copolymers in 1 mL of DMAc at 25 °C.

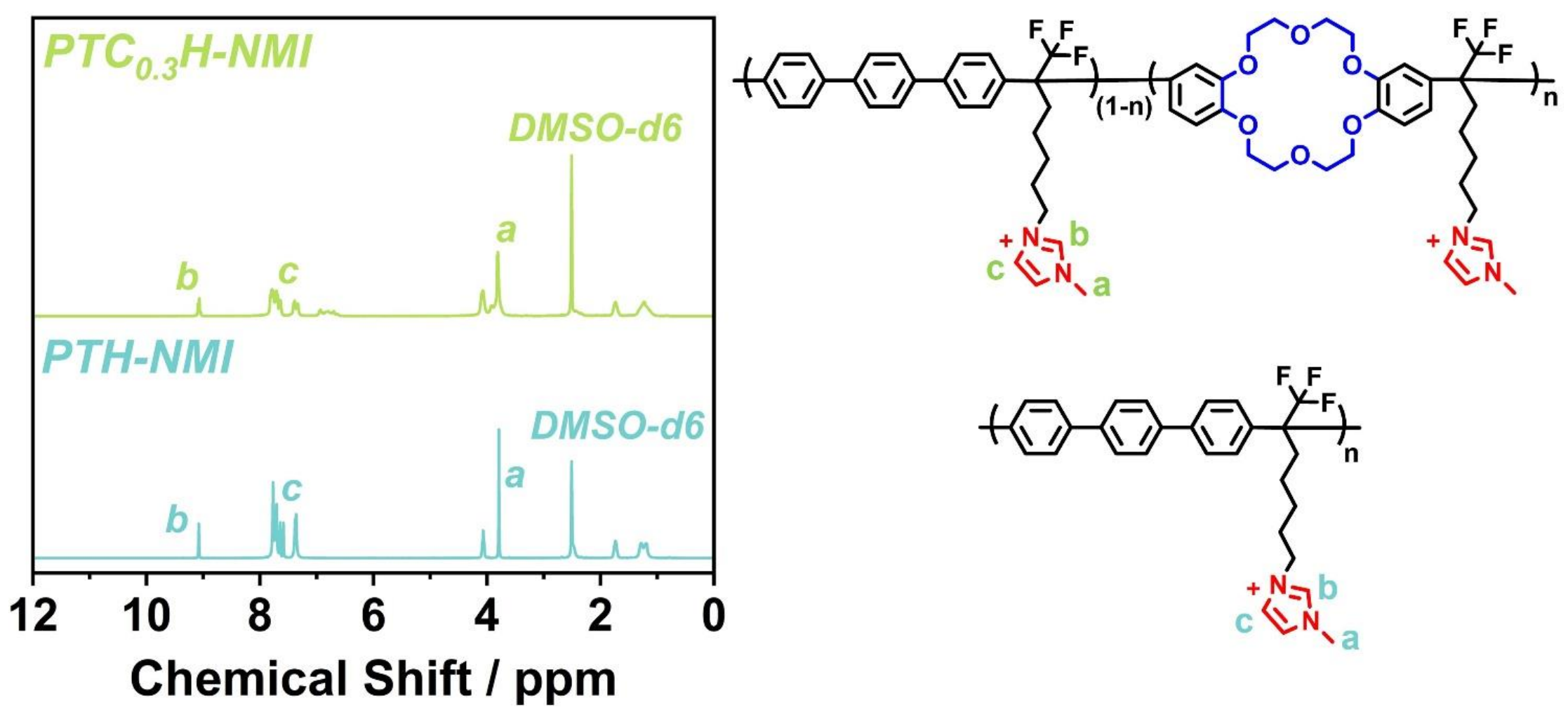

Fig. S7 ^{1}H NMR of PTH-NMI and PTC$_{0.3}$H-NMI.

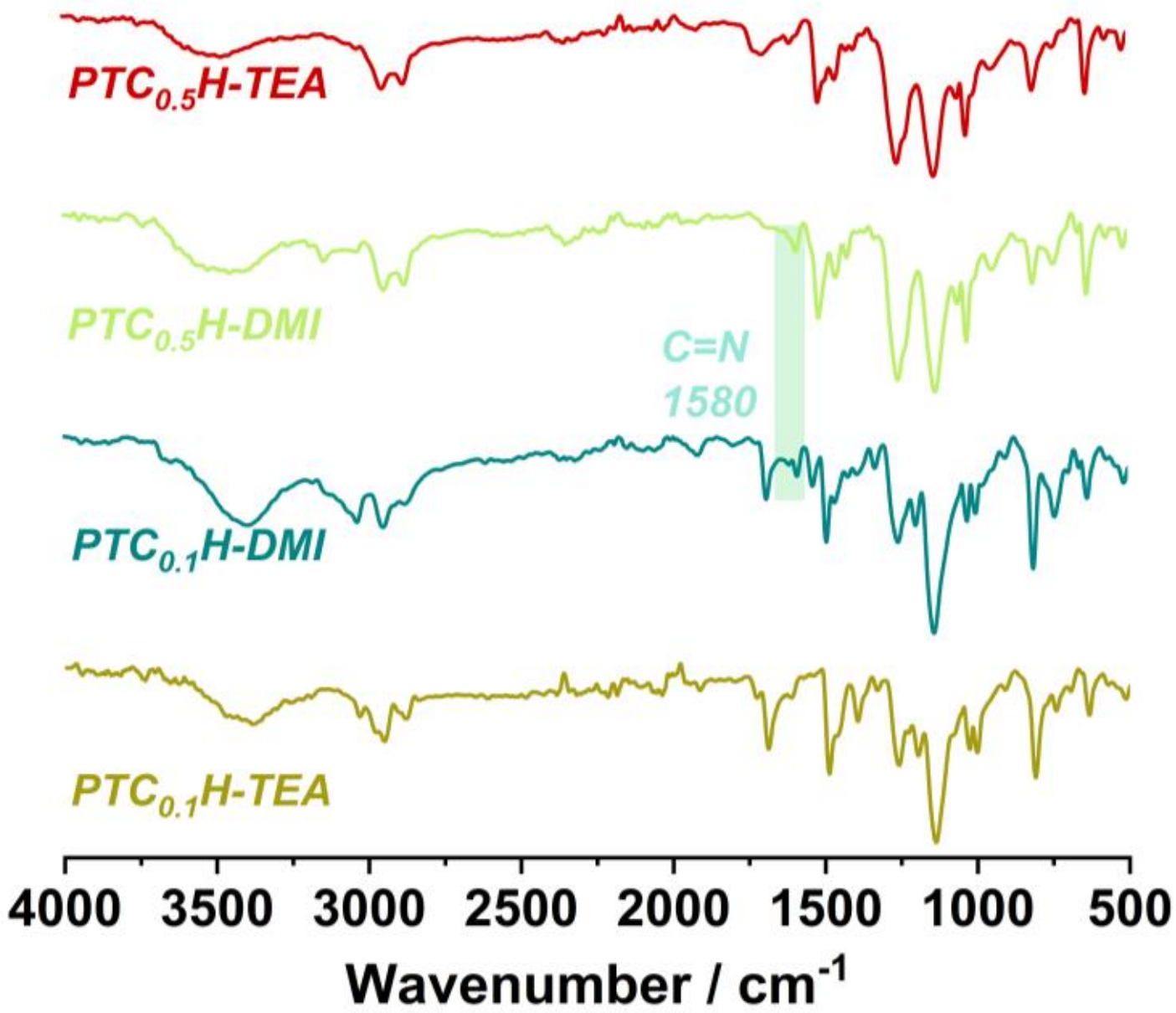

Fig. S8 FTIR of $PTC_{0.5}$H-TEA, $PTC_{0.5}$H-DMI, $PTC_{0.1}$H-DMI and $PTC_{0.1}$H-TEA.

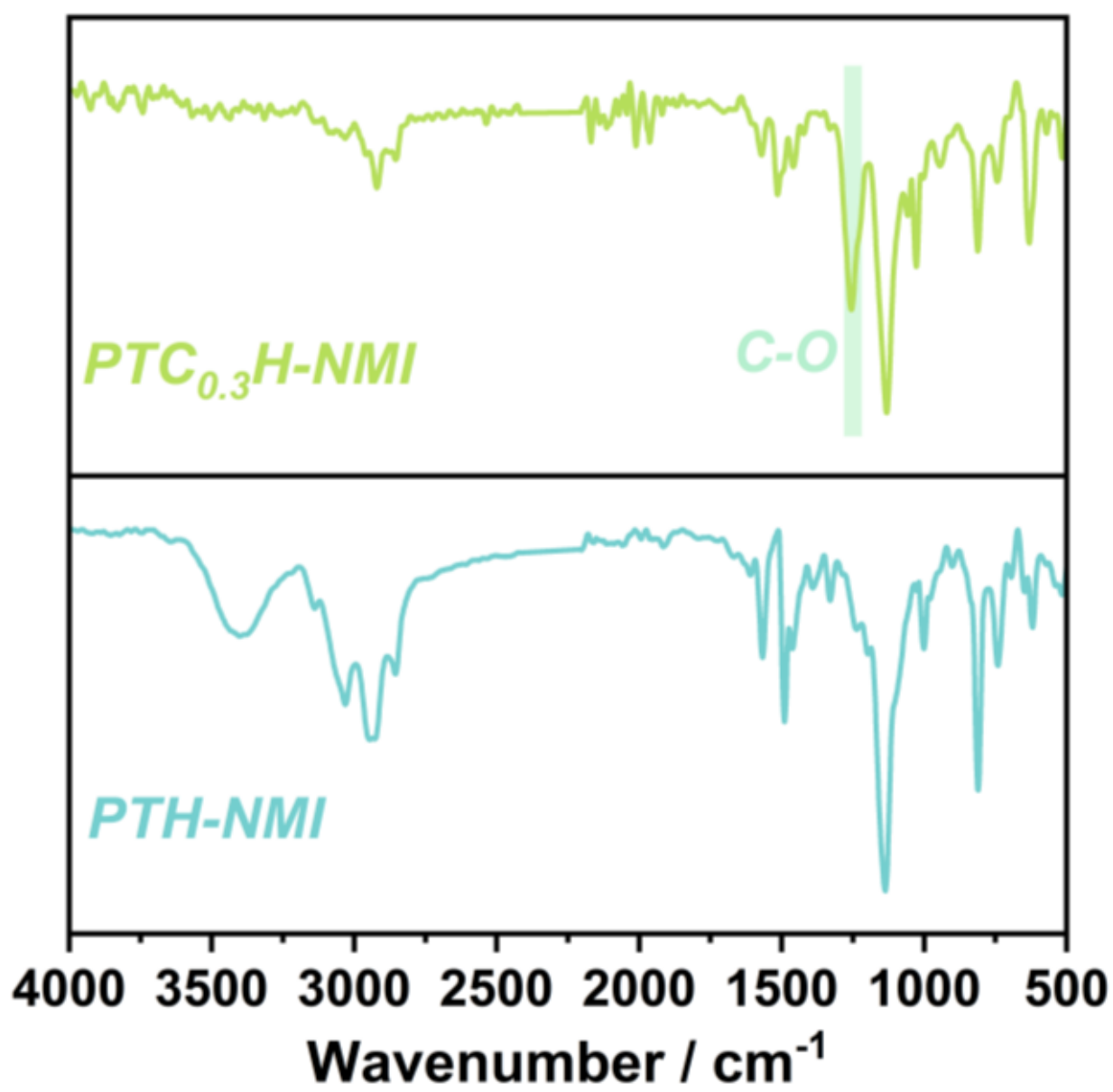

Fig. S9 FTIR of $PTC_{0.3}$H-NMI and PTH-NMI.

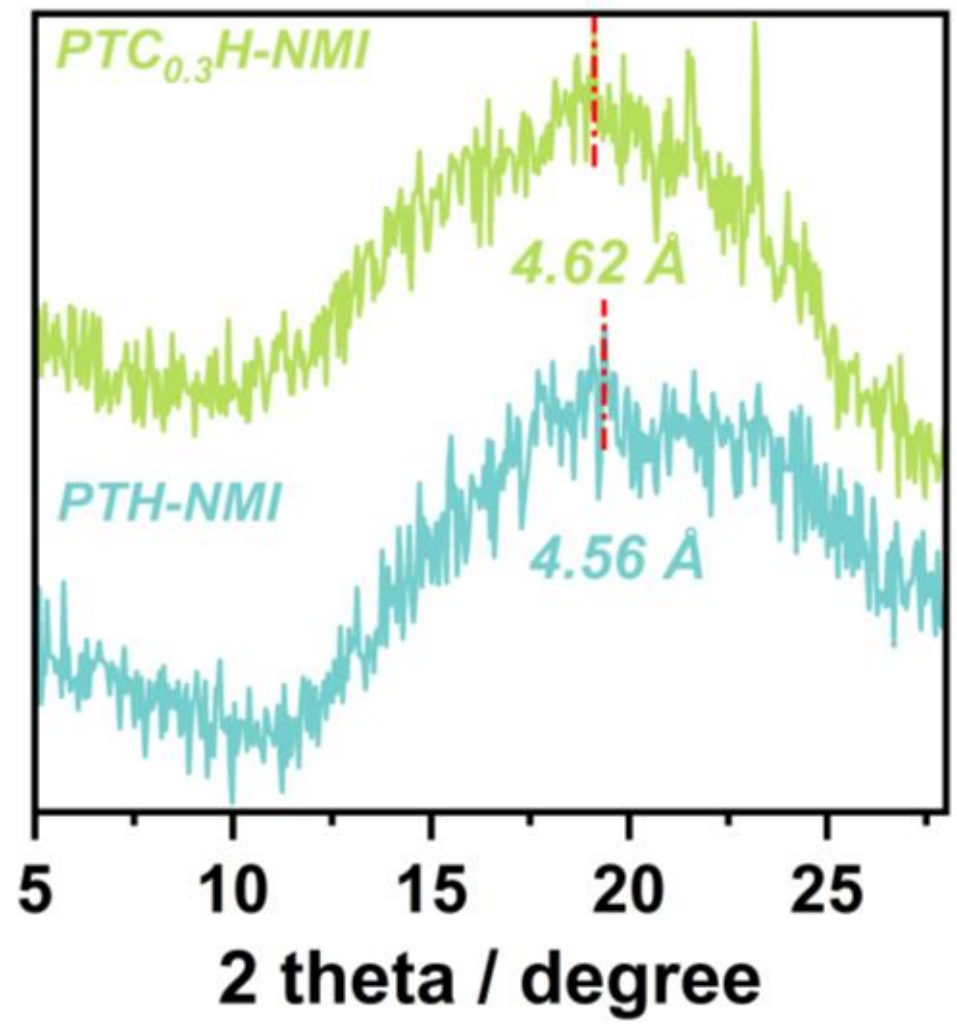

Fig. S10 XRD pattern of $PTC_{0.3}H$-NMI and PTH-NMI.

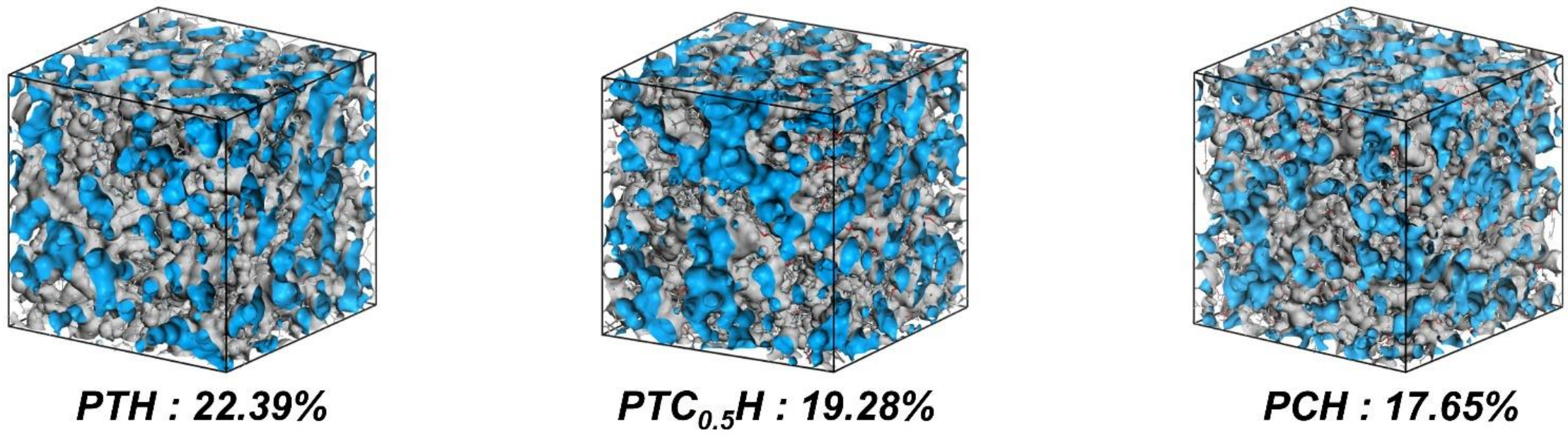

Fig. S11 The MD simulations (accessible volume in blue) of PTH, $PTC_{0.5}H$ and PCH.

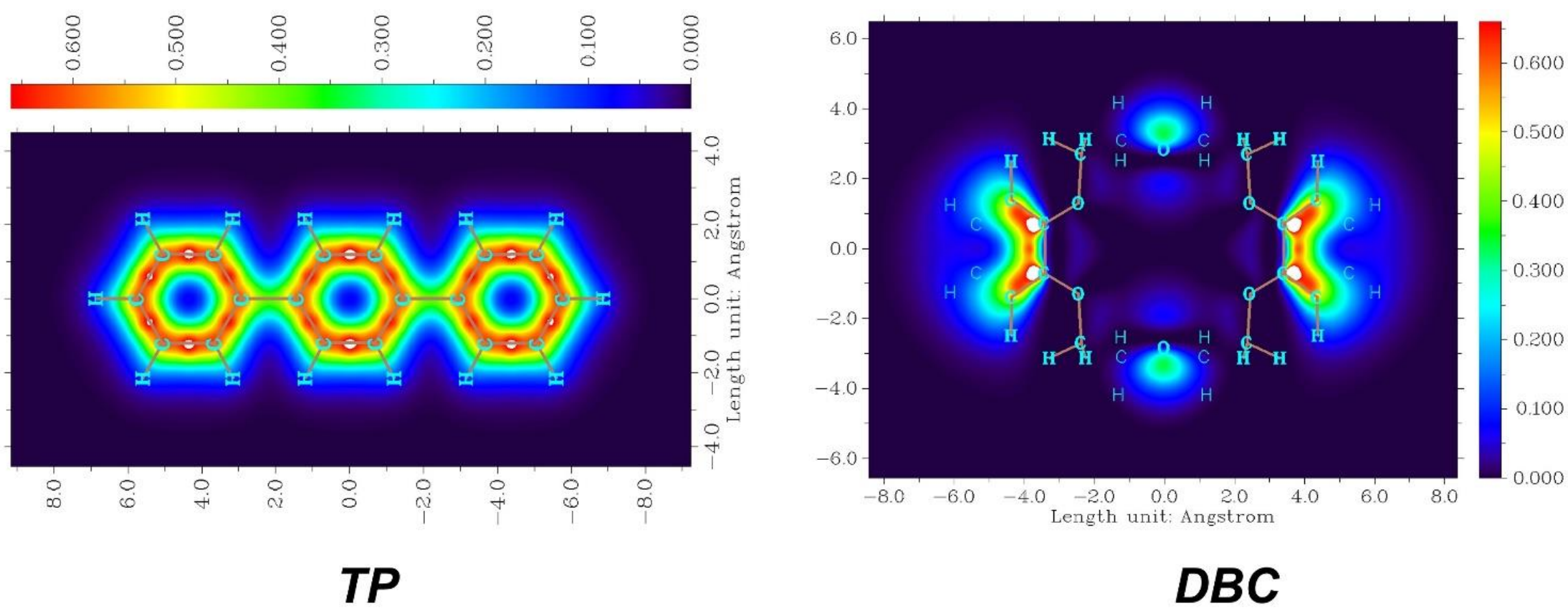

Fig. S12 The color-filled map of the π electron delocalization situation of TP and DBC.

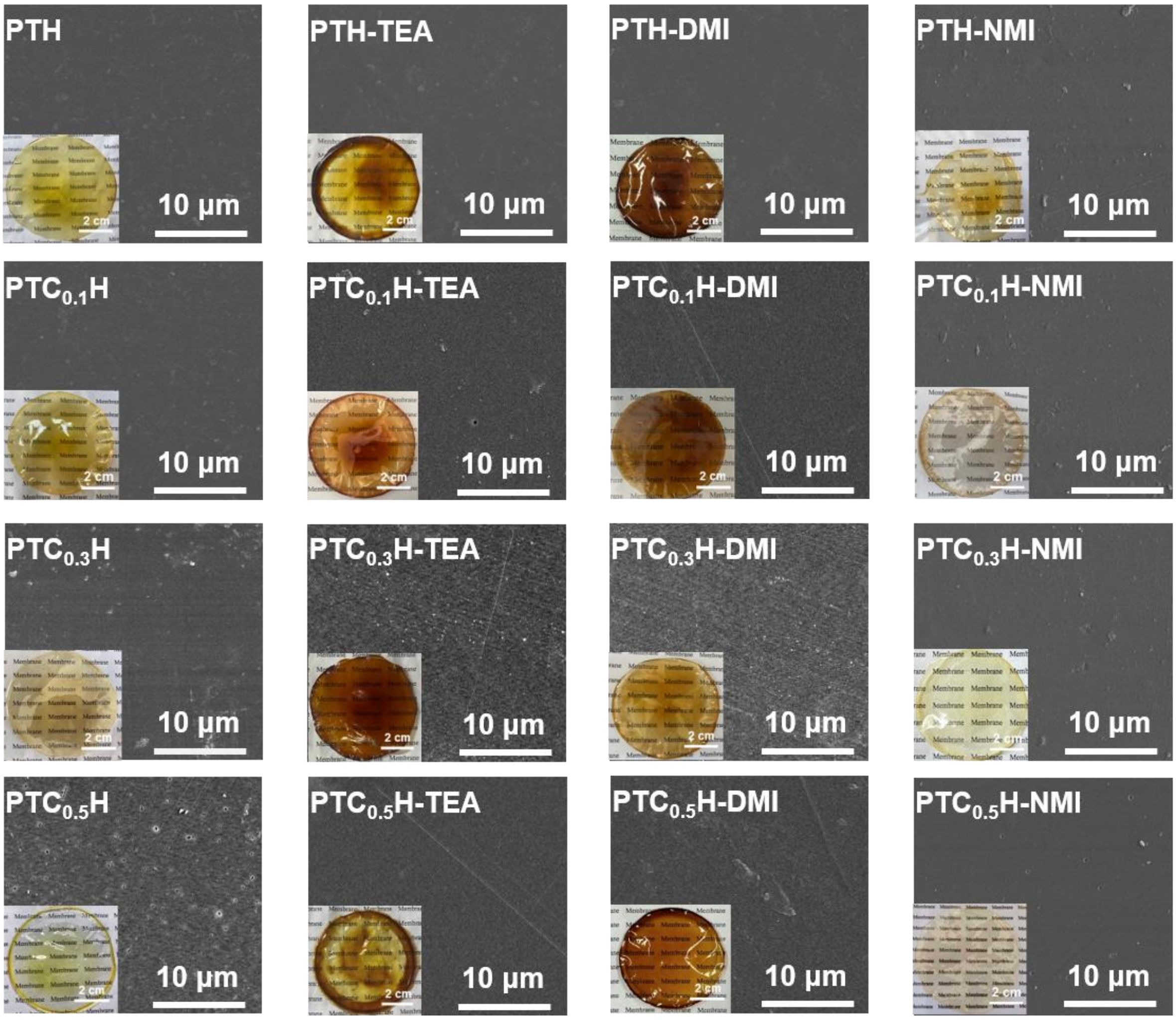

Fig. S13 Surface SEM and digital images of different membranes.

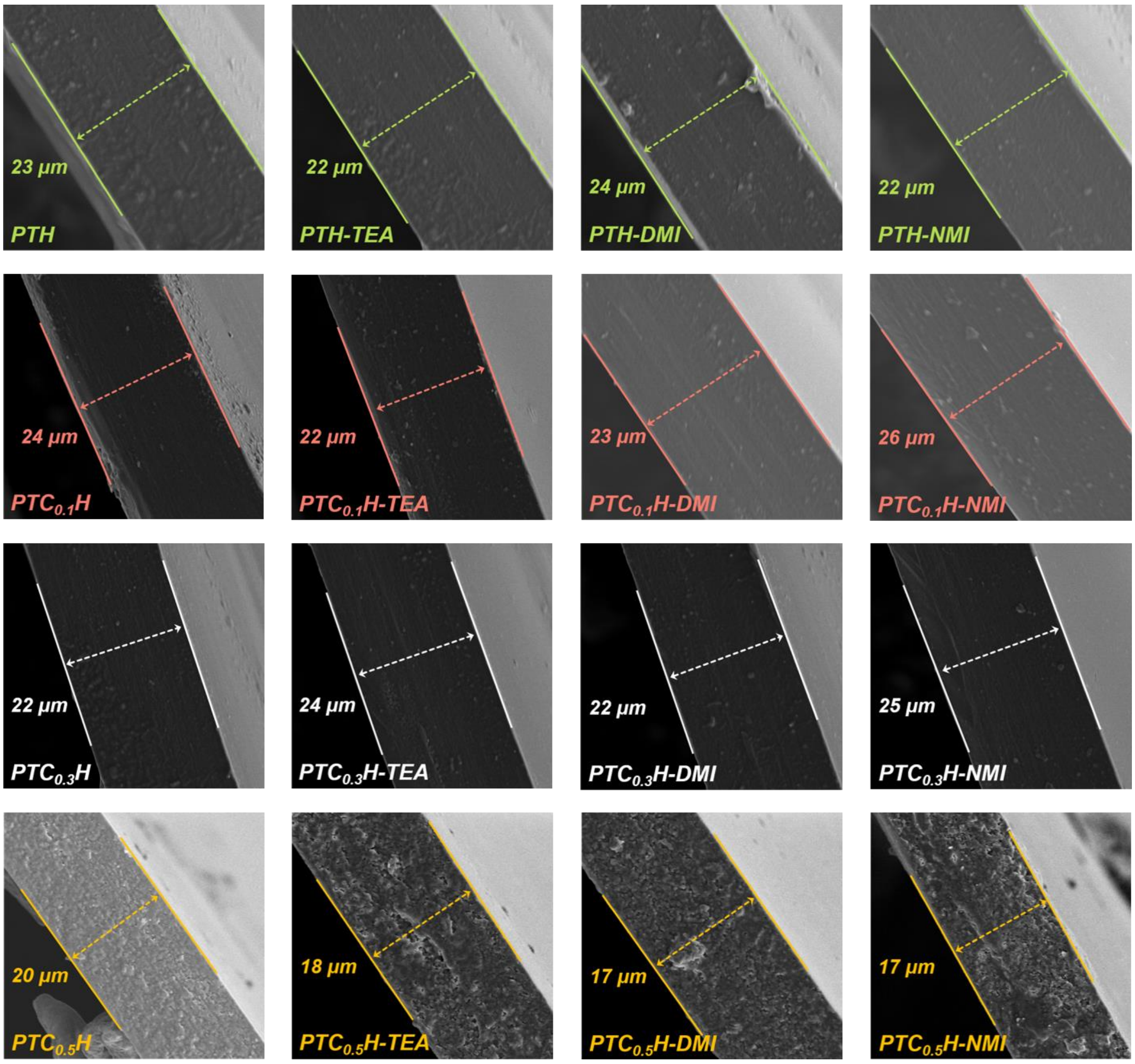

Fig. S14 Cross-sectional SEM images of different membranes.

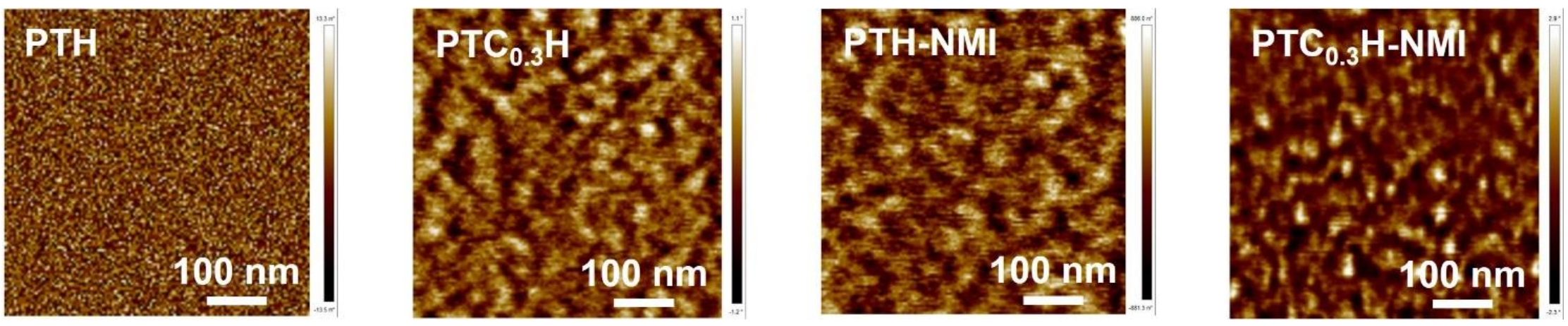

Fig. S15 AFM of different membranes.

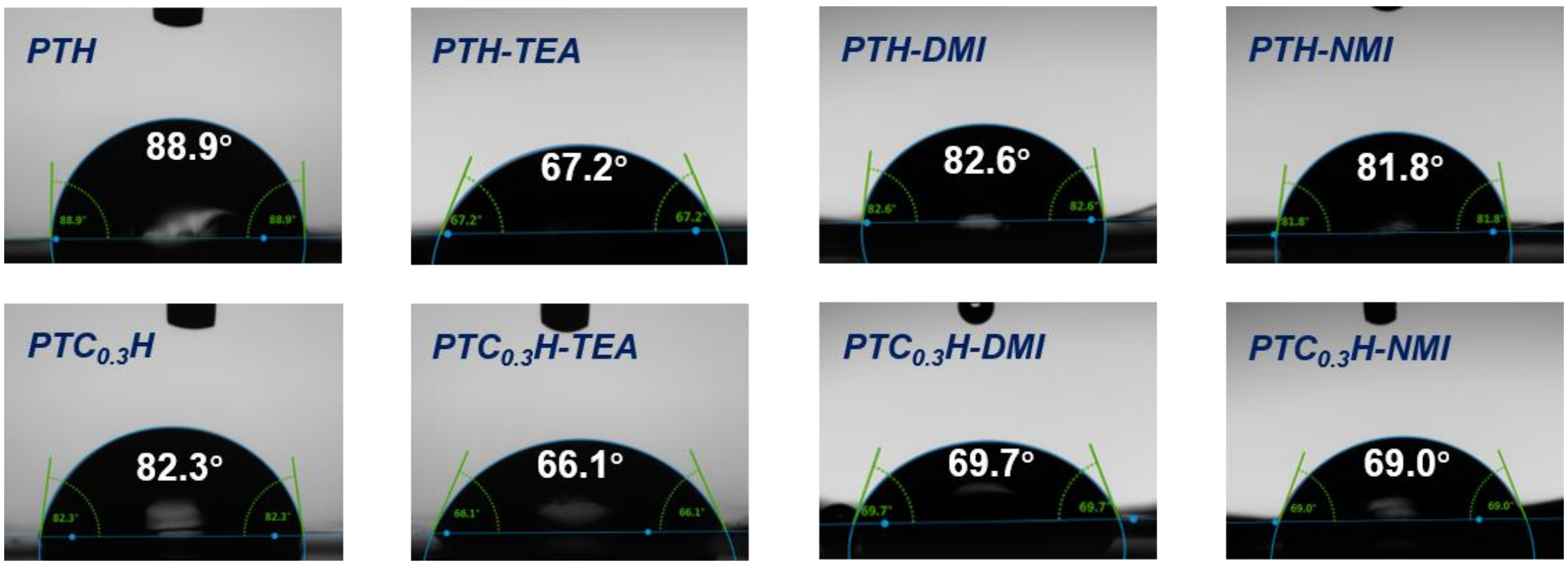

Fig. S16 Water contact angle of different membranes.

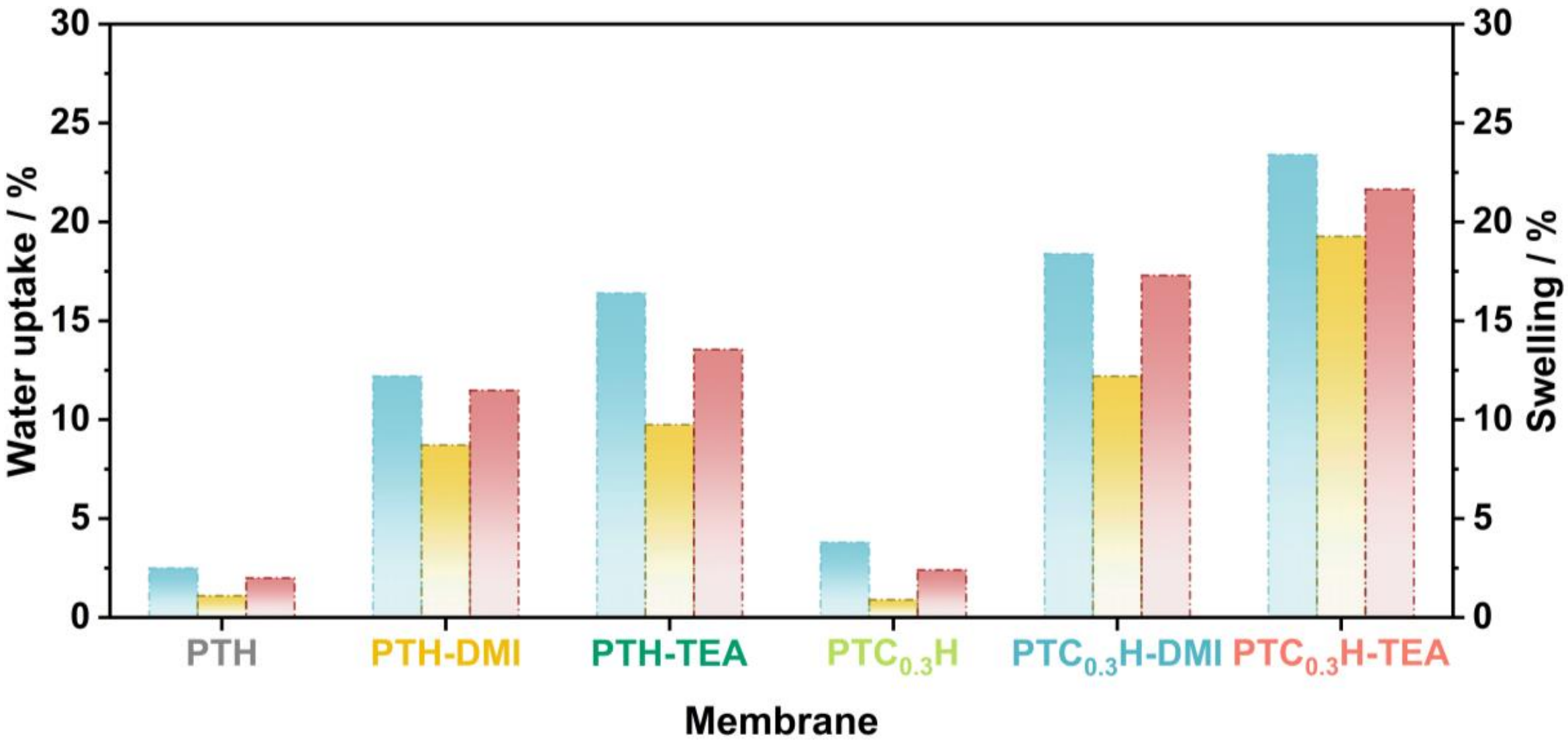

Fig. S17 Water uptake and swellings of different membranes.

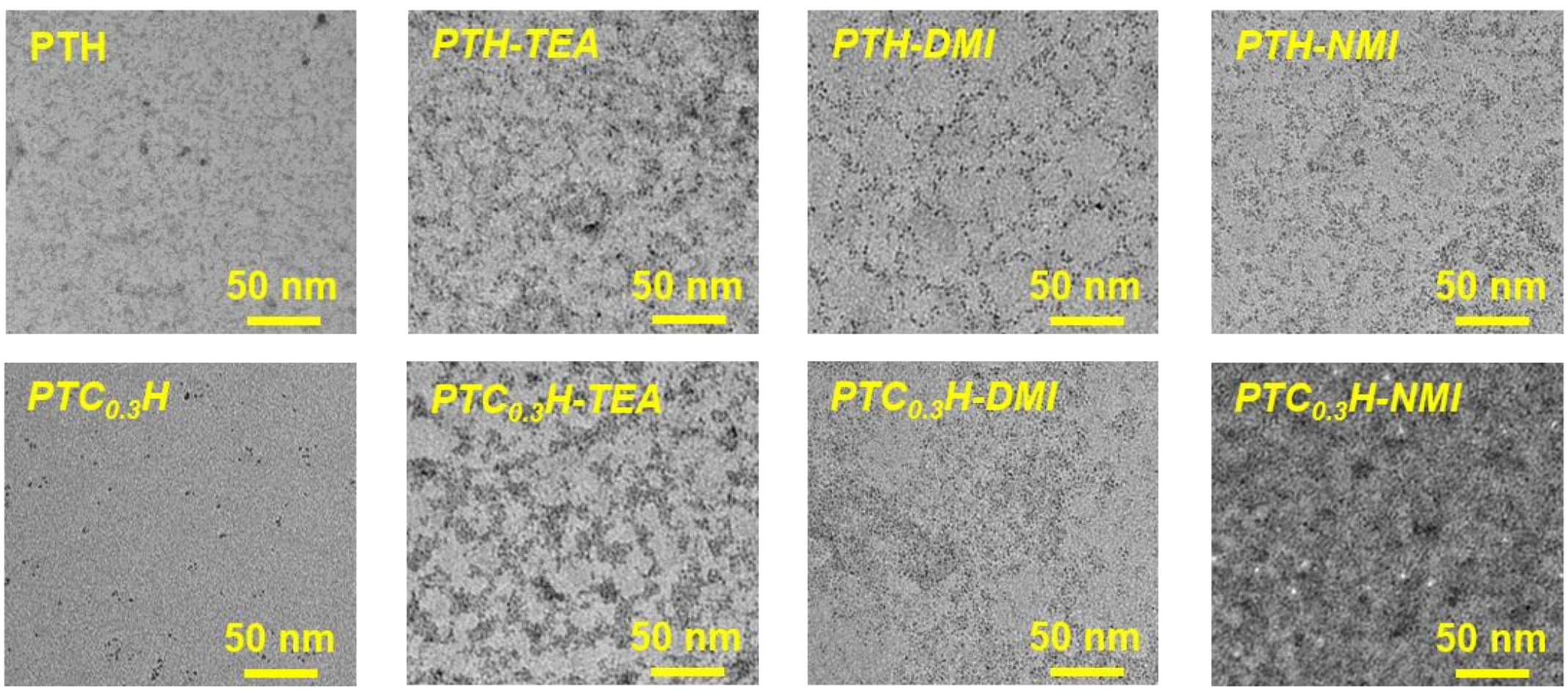

Fig. S18 TEM of different membranes.

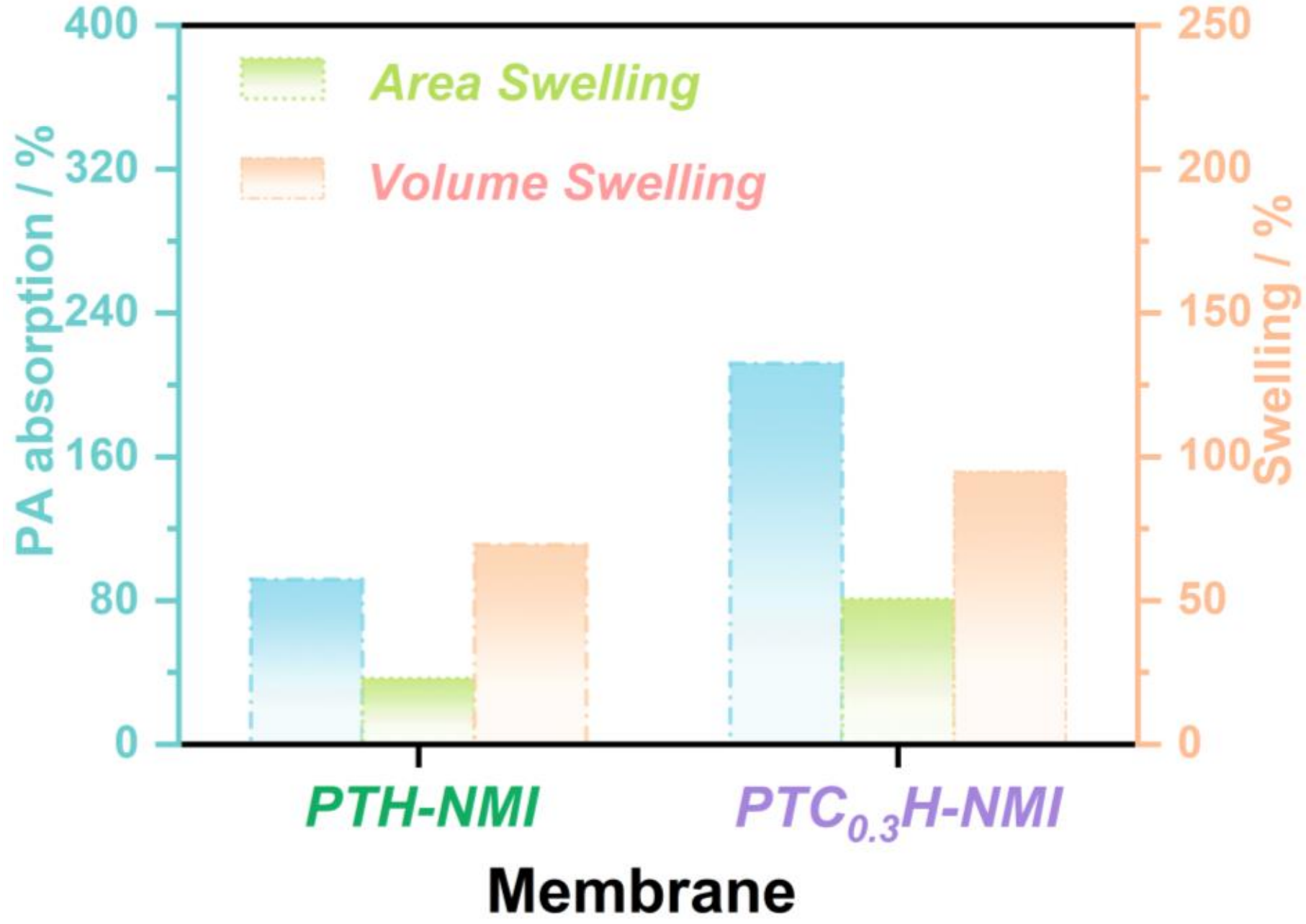

Fig. S19 PA uptake and dimensional change of $PTC_{0.3}H$-NMI and PTH-NMI.

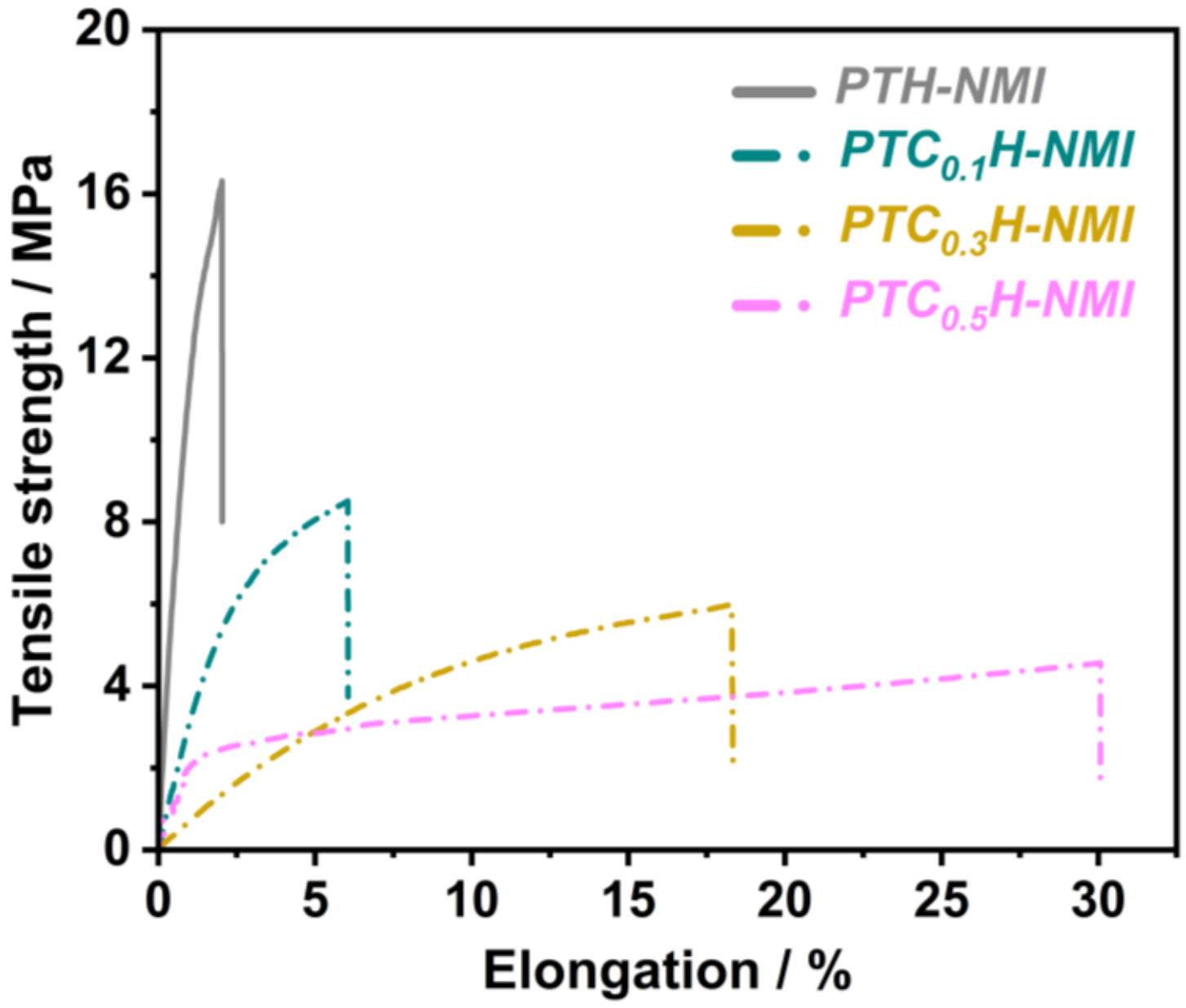

Fig. S20 Mechanical properties of NMI-functionalized series membranes.

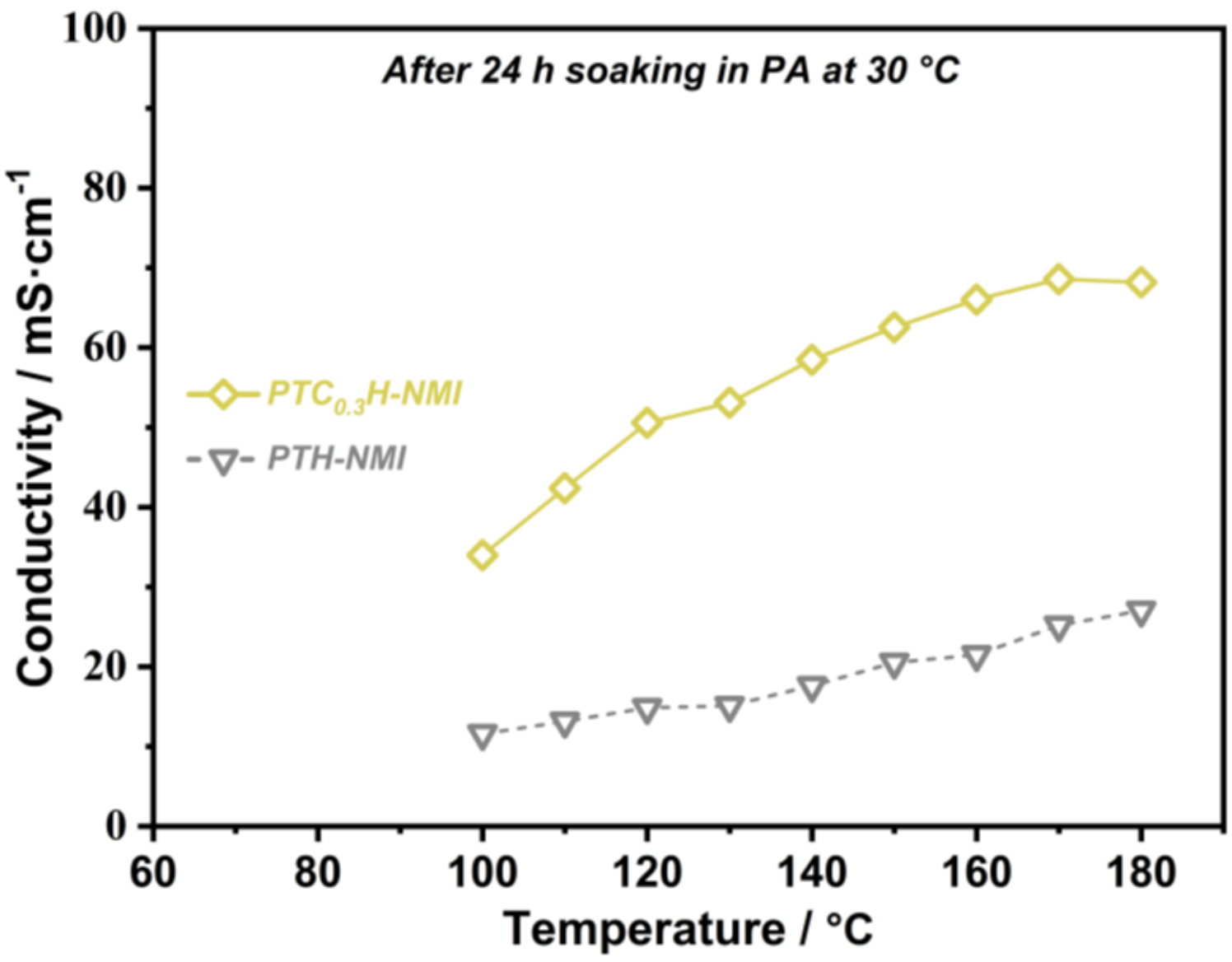

Fig. S21 Anhydrous proton conductivity.

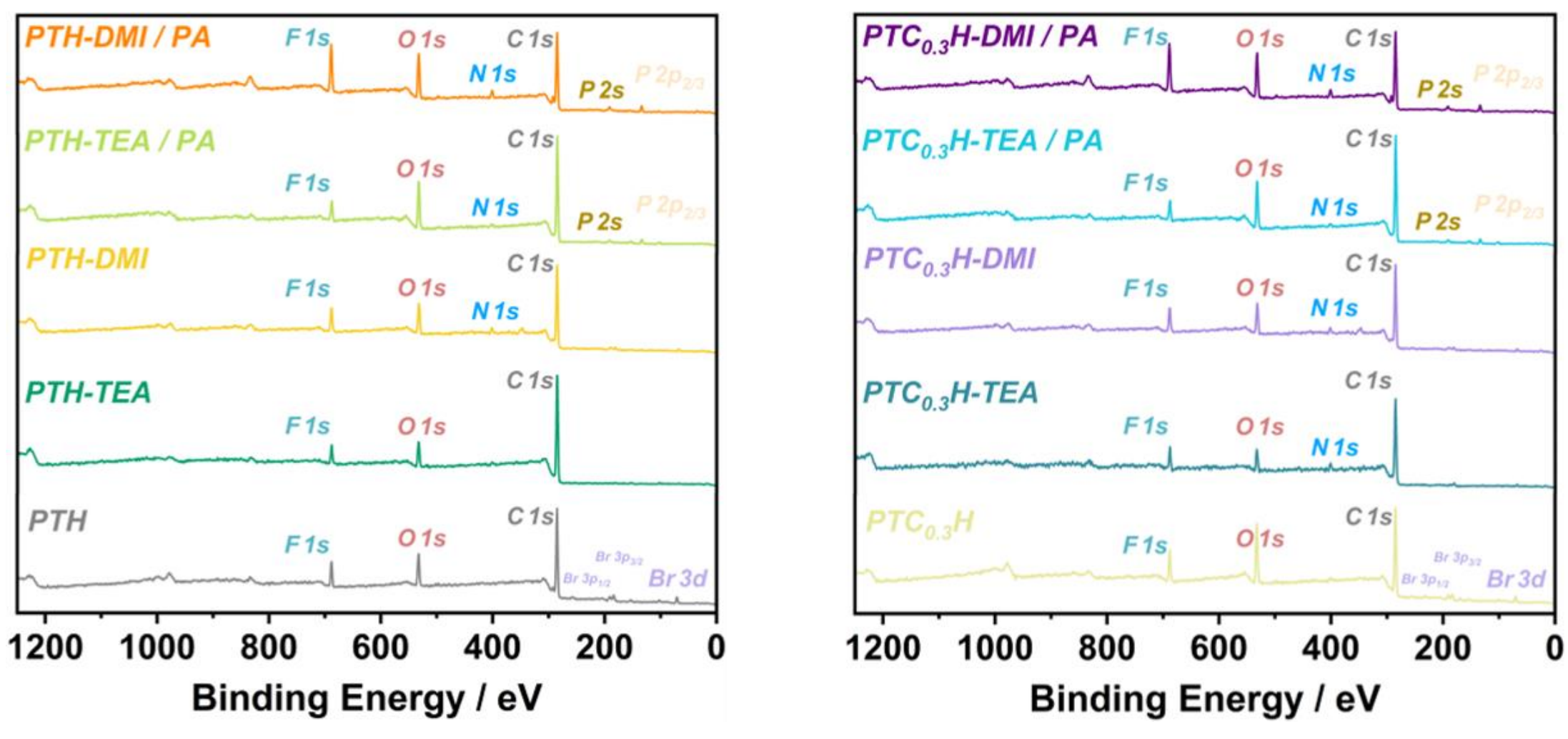

Fig. S22 The full spectrum of XPS for PTH-TEA, PTH-DMI, $PTC_{0.3}$H-TEA and $PTC_{0.3}$H-DMI and after doping with PA.

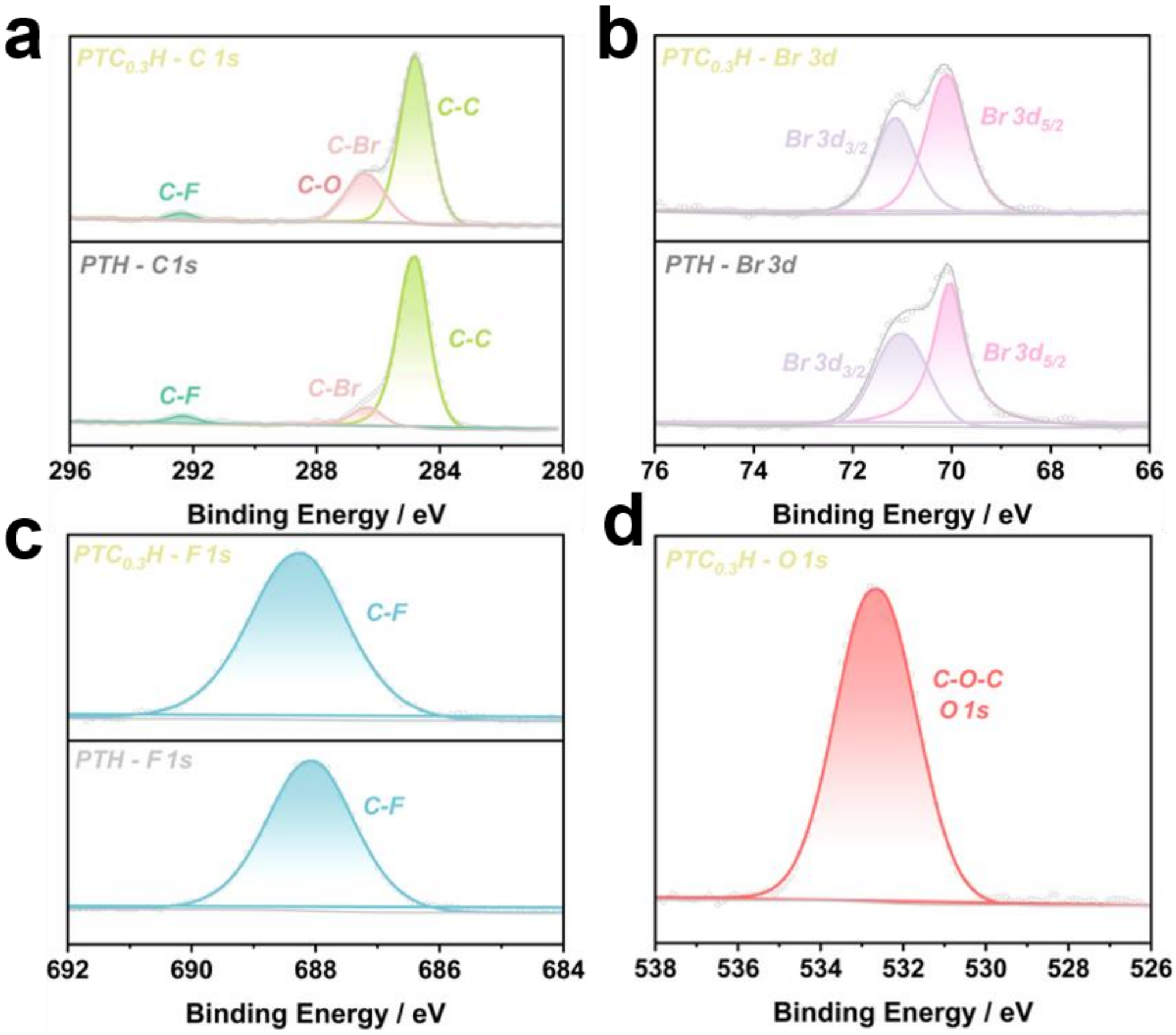

Fig. S23 (a) The XPS-C1s, (b) XPS-Br 3d, (c) XPS-F1s, (d) XPS-O1s for PTH and $PTC_{0.3}H$.

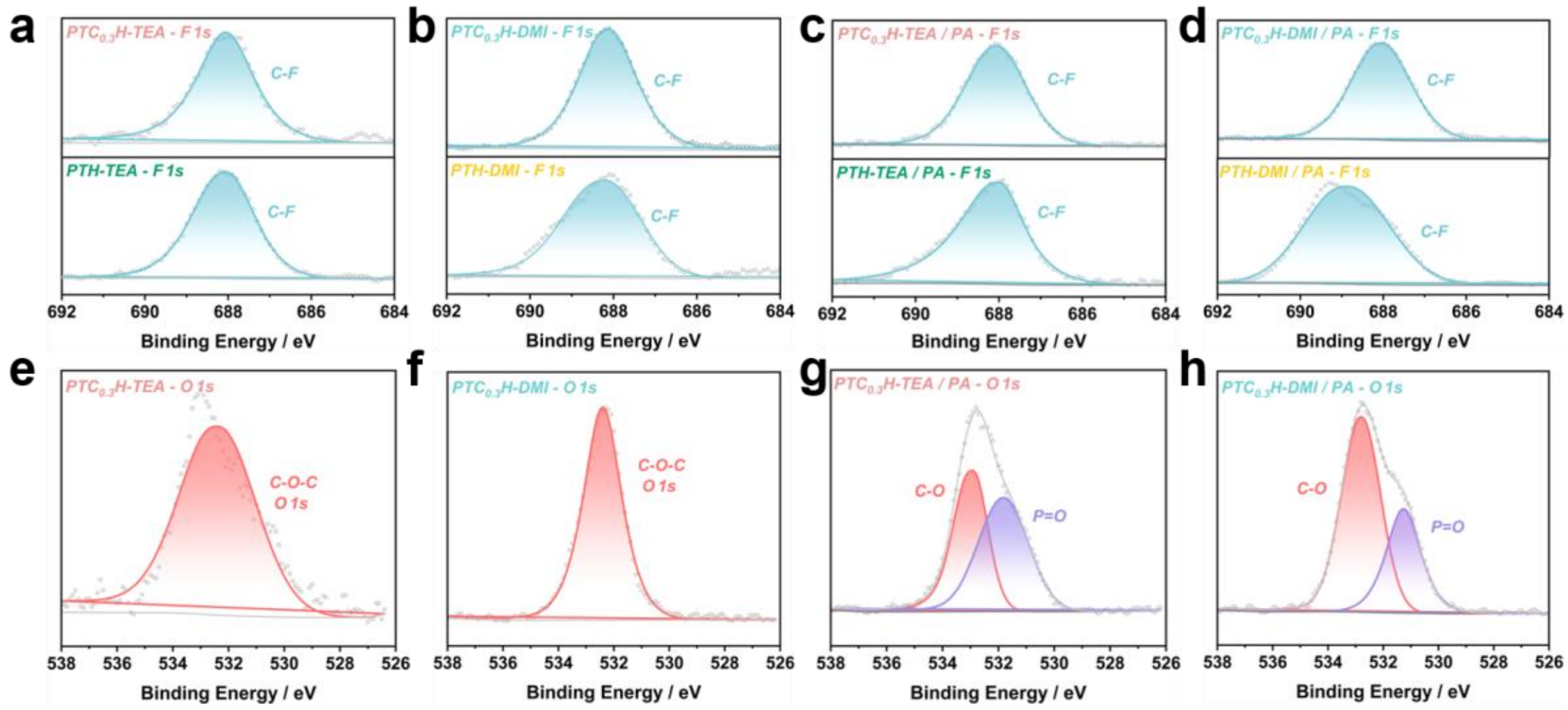

Fig. S24 (a-d) The XPS-F1s, (e-h) XPS-O1s of PTH-TEA, PTH-DMI, $PTC_{0.3}H$-TEA and $PTC_{0.3}H$-DMI and after doping with PA.

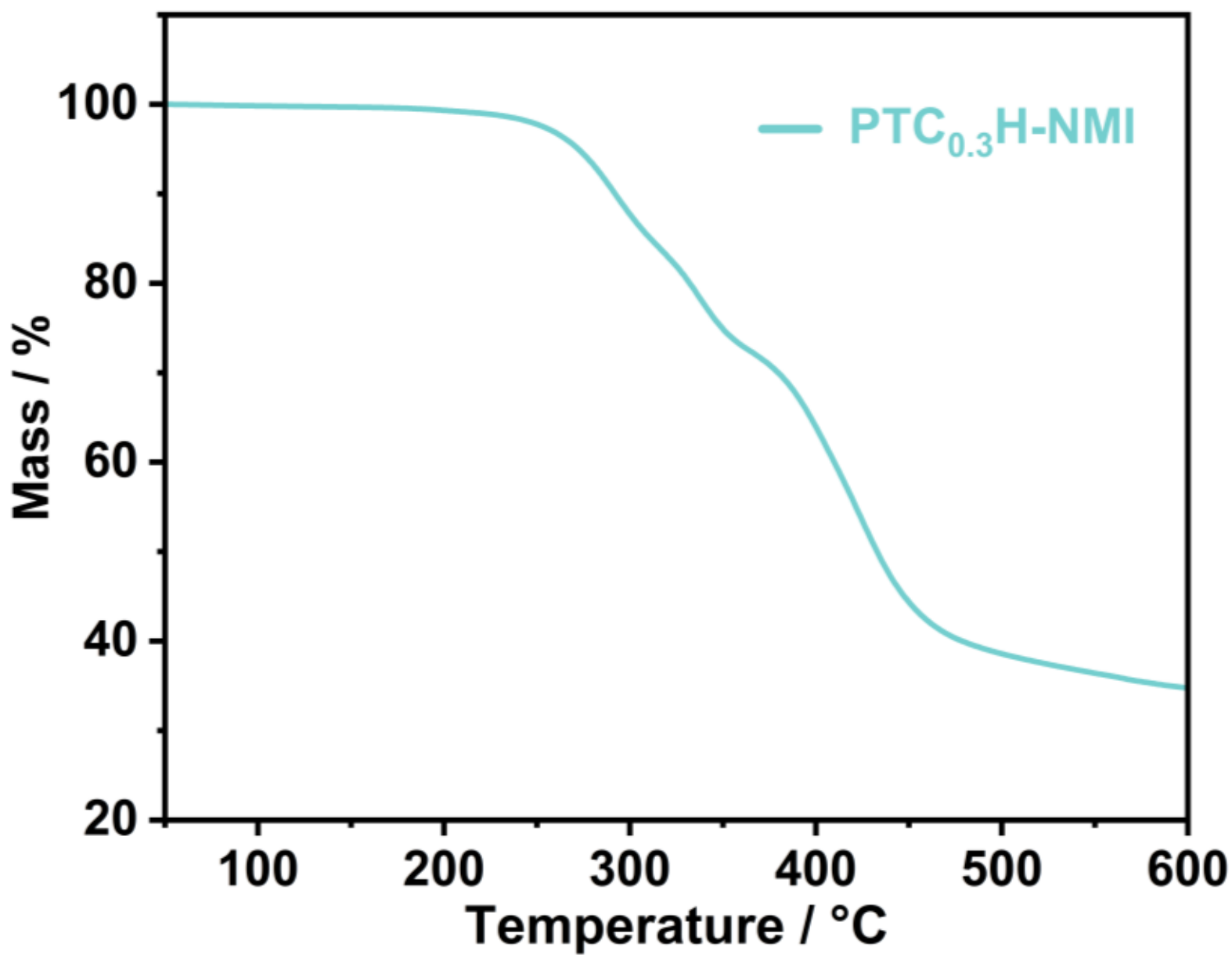

Fig. S25 TGA curve of $PTC_{0.3}H$-NMI.

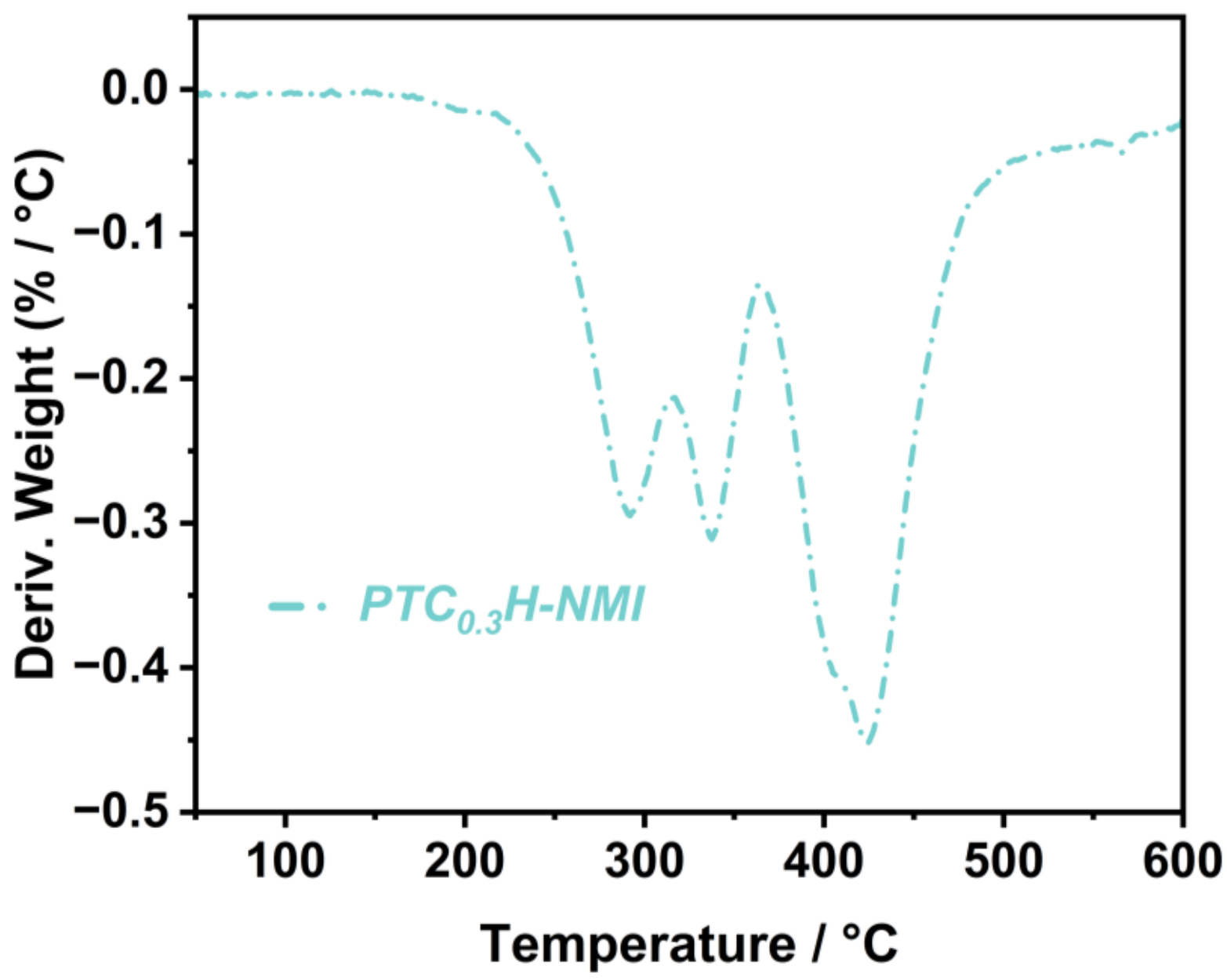

Fig. S26 DTG curve of $PTC_{0.3}H$-NMI.

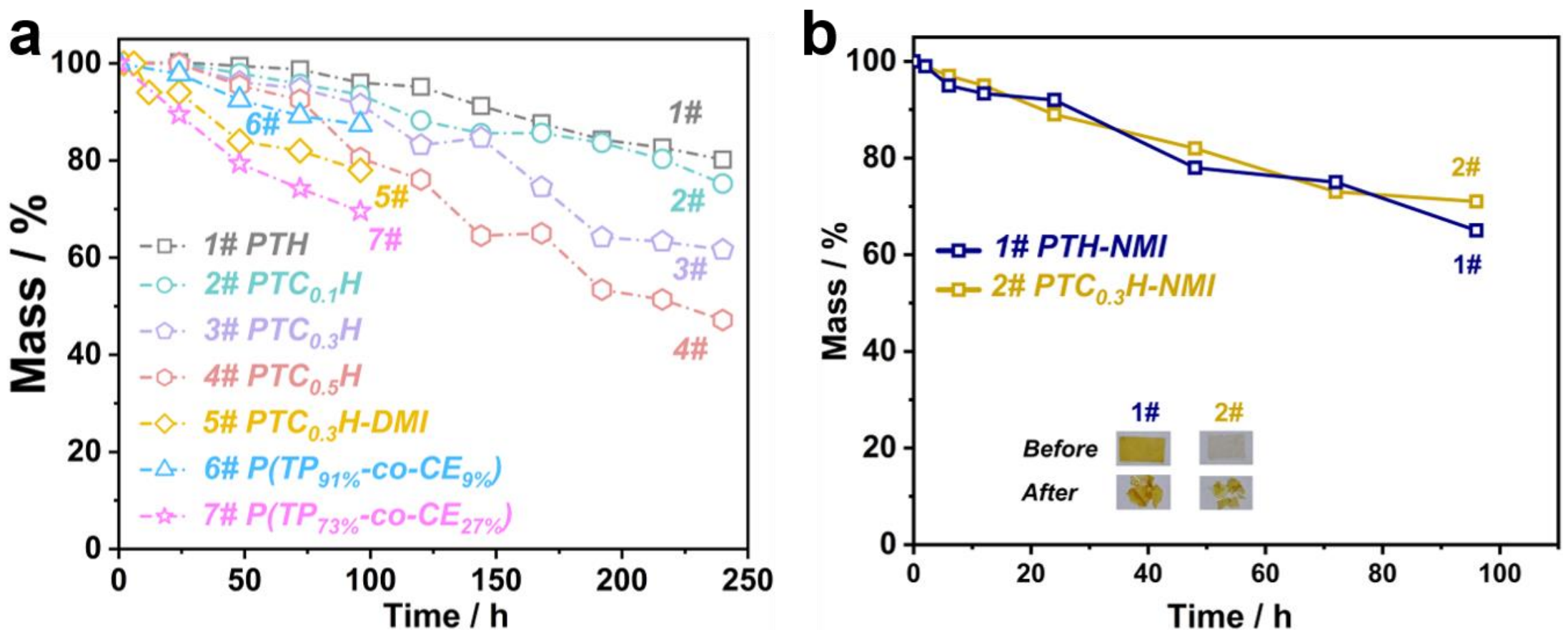

Fig. S27 (a) Fenton test (in 3% H_2O_2 and 3 ppm Fe^{2+} at 80 °C) results of pure PTH and PTC_nH membranes, along with $PTC_{0.3}$H-DMI and P($TP_{91\%}$-co-$CE_{9\%}$) (in our previous work). (b) *Fenton test results (in 3% H_2O_2 and 3 ppm Fe^{2+} at 80 °C) including digital photos of PTH-NMI and $PTC_{0.3}$H-NMI before and after Fenton test.*

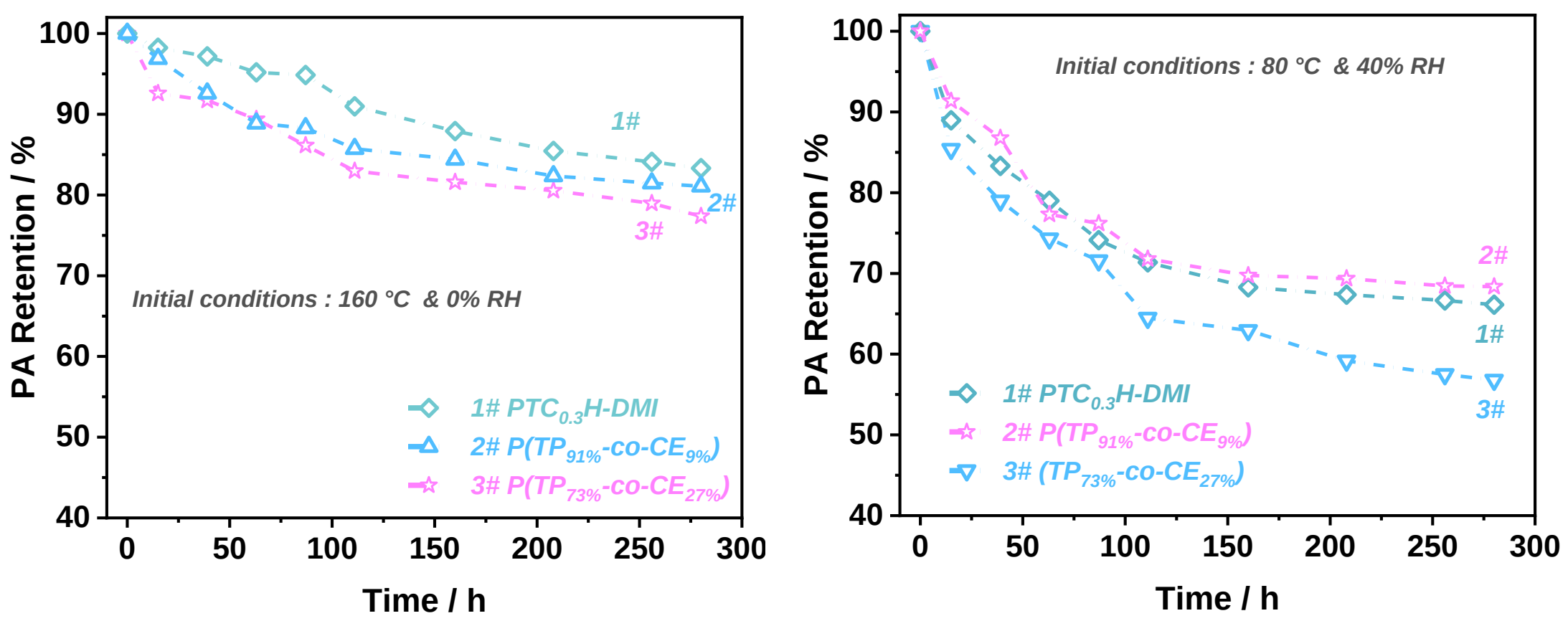

Fig. S28 PA retention of $PTC_{0.3}$H-DMI/PA P($TP_{73\%}$-co-$CE_{27\%}$)/PA and P($TP_{91\%}$-co-$CE_{9\%}$)/PA membranes under the same conditions.

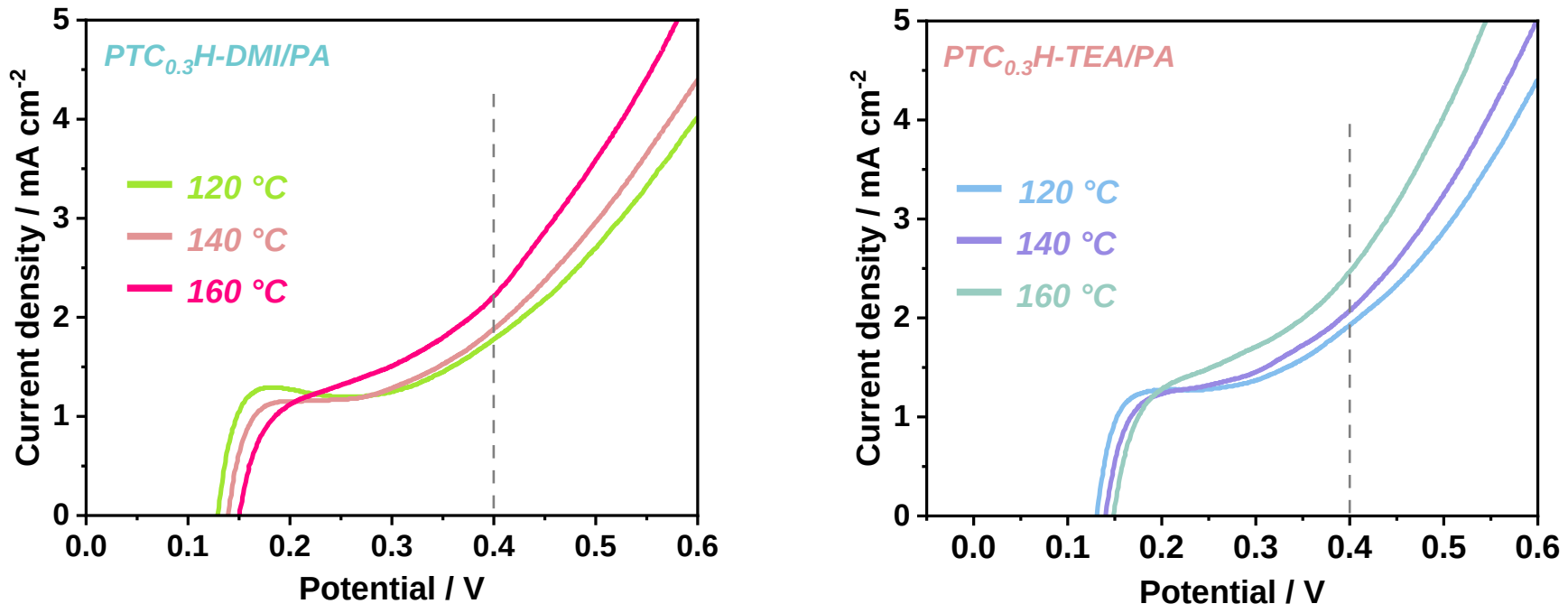

Fig. S29 LSV curves of cells with $PTC_{0.3}$H-DMI/197.2%PA and $PTC_{0.3}$H-TEA/227.5%PA at different temperatures.

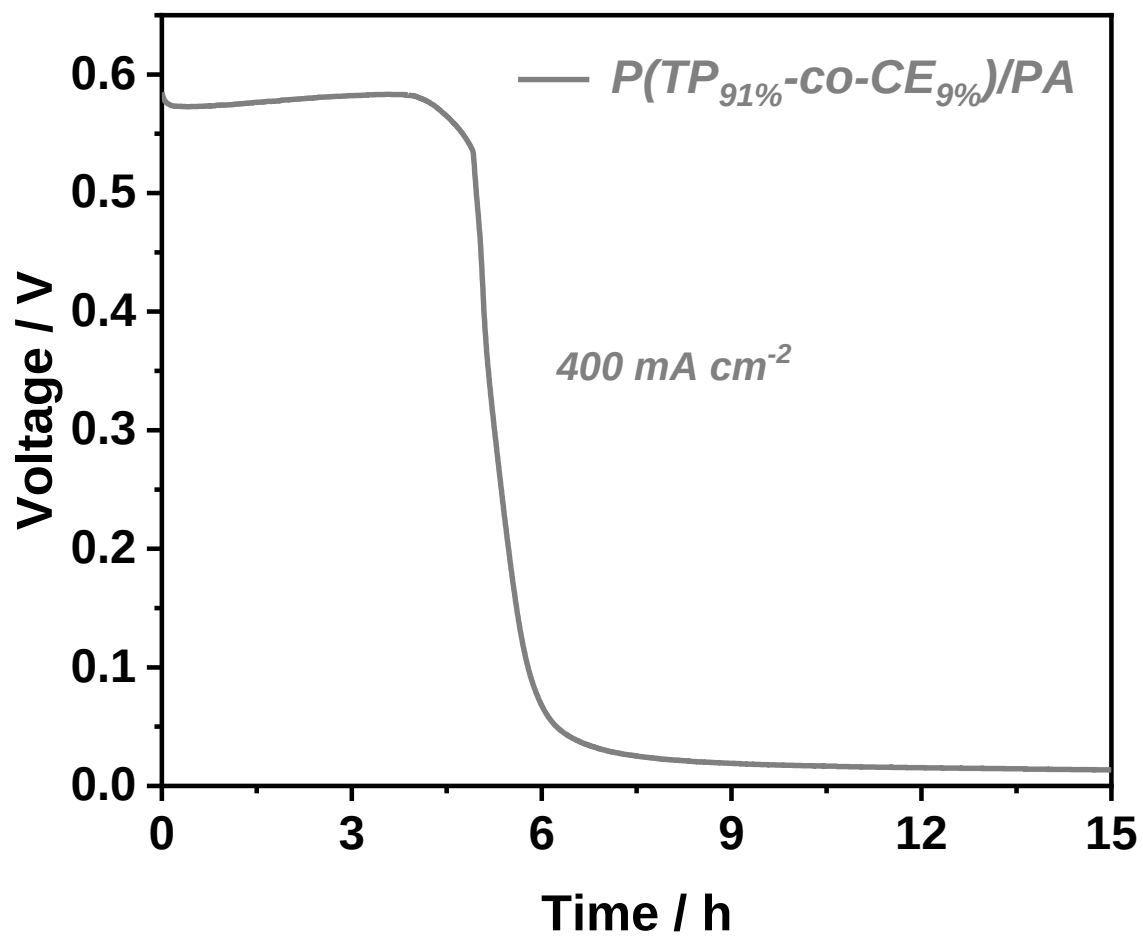

Fig. S30 Long-term operational stability of the cell with P($TP_{91\%}$-co-$CE_{9\%}$) at 160 °C under 400 mA cm^{-2}.

Supplementary tables

Table S1 Comparisons of $PTC_{0.3}H$-DMI in this work and $P(TP_{91\%}$-co-$CE_{9\%})$ in previous work (Science China Chemistry, 2025, 68(3), 1078-1090)

Properties	Membrane	
	$PTC_{0.3}H$-DMI	$P(TP_{91\%}$-co-$CE_{9\%})$
ADC%	197	205
PA retention (160°C and 0%RH)	83%	81%
Chemical stability (Fenton test at 80°C)	80%	87%
Conductivity at 180 °C / mS cm^{-1}	65.3	138
Tensile strength at RT / MPa	5.9	7.5
Elongation at RT / %	12.5	9.2
H_2/O_2 peak power density at 180 °C / mW cm^{-2}	1152	1194
Durability at 400 mA cm^{-2}/ h	42	<5

Table S2. The detailed values of energy decomposition (kcal mol^{-1}) for TP-PA and DBC-PA.

Molecule	E_{total}	E_{els}	E_x	E_{rep}	E_{orb}	E_{DFTc}	E_{dc}
TP-PA	-9.62	-9.69	-6.63	27.82	-6.11	-6.35	-8.67
DBC-PA	-24.87	-35.4	-20.7	79.27	-17.21	-15.89	-14.94

Table S3. Testing conditions of HT-PEMFCs in this work and literature.

**Membrane**	**PA uptake / %**	**Conductivity / mS cm^{-1}**	**Anode**	**Cathode**	**P_{peak} / mW cm^{-2}**	**Reference**
$PTC_{0.3}H$-DMI	197.2	65.31	Pt (0.9 mg cm^{-2})	Pt (0.9 mg cm^{-2})	1152	This work
$PTC_{0.3}H$-TEA	227.5	65.08	Pt (0.9 mg cm^{-2})	Pt (0.9 mg cm^{-2})	785	This work
PQBN-4-IM (bp)	379.9	90.26	Pt/C (0.5 mg cm^{-2})	Pt/C (0.5 mg cm^{-2})	802.5	[5]

St(100)-OPBI-GO	4.65/ADL	128	———	———	422.31	[6]
P(TP-4TA)	158	52.2	Pt/C (1 mg cm^{-2})	PtCo/C (1 mg cm^{-2})	684	[7]
PTP-0.21BmBZ	315	112	Pt (0.6 mg cm^{-2})	Pt (0.6 mg cm^{-2})	1014	[8]
PTP-co-PBP2F15-IM	285.89	145.11	Pt (1 mg cm^{-2})	Pt (1 mg cm^{-2})	1019	[9]
b-PTTP-BIm-DP20	126	167	Pt (0.6 mg cm^{-2})	Pt (0.6 mg cm^{-2})	835	[10]
PTAP	220	102	Pt/C (1 mg cm^{-2})	Pt/C (1 mg cm^{-2})	743	[11]
60-40-QOH	227	85.42	———	———	529	[12]
Blend7/3	292	409	Pt (1 mg cm^{-2})	Pt (0.7 mg cm^{-2})	1060	[13]
OPBI	162	21.39	Pt/C (0.9 mg cm^{-2})	Pt/C (0.9 mg cm^{-2})	437	[14]
ABPBI-0.5EGO	2.3/ADL	47.02	———	———	349	[15]
PBFI-86% GTA	324	90	Pt/C (0.9 mg cm^{-2})	Pt/C (0.9 mg cm^{-2})	876	[14]
PTP-10cPIM	149.26	107.91	Pt (1 mg cm^{-2})	Pt (1 mg cm^{-2})	766	[16]
tPTP-2	269	137.5	Pt/C (0.6 mg cm^{-2})	Pt/C (0.6 mg cm^{-2})	655.4	[17]
P(TP91%-co-CE9%)	205	138	Pt/C (0.9 mg cm^{-2})	Pt/C (0.9 mg cm^{-2})	1194	[18]
DMBP-TB/50%pEKC	149	61	Pt/C (0.7 mg cm^{-2})	Pt/C (0.7 mg cm^{-2})	536	[19]
QPAF-225-10 (200)	2.83/ADL	174	Pt (0.3 mg cm^{-2})	Pt (0.3 mg cm^{-2})	847 (200 °C)	[20]
PTC$_{0.3}$H-DMI	197.2	62.35	Pt (0.9 mg cm^{-2})	Pt (0.9 mg cm^{-2})	1104 (160 °C)	This work
PTC$_{0.3}$H-TEA	227.5	69.25	Pt (0.9 mg cm^{-2})	Pt (0.9 mg cm^{-2})	683(160 °C)	This work
TP-2-IM (160)	401	66	Pt/C (0.5 mg cm^{-2})	Pt/C (0.5 mg cm^{-2})	621(160 °C)	[21]
COF-BQA(3%)/OPBI	223.4	88	Pt/C (0.8 mg cm^{-2})	Pt/C (0.8 mg cm^{-2})	615.5(160 °C)	[22]
PP70-EDTMPA	160	120	Pt/C (1 mg cm^{-2})	Pt/C (1 mg cm^{-2})	994 (160 °C)	[23]
PTP-10CPIM	149.26	92.5	Pt (1 mg cm^{-2})	Pt 1 mg cm^{-2})	694 (160 °C)	[16]
P-mPBI-1/9	475.8	86	Pt (0.5 mg cm^{-2})	Pt (0.5 mg cm^{-2})	586 (160 °C)	[24]
mPBI	397	73	Pt (0.5 mg cm^{-2})	Pt (0.5 mg cm^{-2})	481 (160 °C)	[24]

CBOPBI-P40	296	84	Pt (1 mg cm^{-2})	Pt (1 mg cm^{-2})	699 (160 °C)	[25]
OPBI-PIL-30%	344.9	126.7	Pt (1 mg cm^{-2})	Pt (2 mg cm^{-2})	449 (160 °C)	[26]
OPBI&P-phIm-10%	222.22	117	Pt/C (0.8 mg cm^{-2})	Pt/C (0.8 mg cm^{-2})	568 (160 °C)	[27]

References

[1]. Gaussian 16, A.03, M. J. Frisch, G. W. Trucks, H. B. Schlegel, G. E. Scuseria, M. A. Robb, J. R. Cheeseman, G. Scalmani, V. Barone, G. A. Petersson, H. Nakatsuji, X. Li, M. Caricato, A. V. Marenich, J. Bloino, B. G. Janesko, R. Gomperts, B. Mennucci, H. P. Hratchian, J. V. Ortiz, A. F. Izmaylov, J. L. Sonnenberg, Williams, F. Ding, F. Lipparini, F. Egidi, J. Goings, B. Peng, A. Petrone, T. Henderson, D. Ranasinghe, V. G. Zakrzewski, J. Gao, N. Rega, G. Zheng, W. Liang, M. Hada, M. Ehara, K. Toyota, R. Fukuda, J. Hasegawa, M. Ishida, T. Nakajima, Y. Honda, O. Kitao, H. Nakai, T. Vreven, K. Throssell, J. A. Montgomery Jr., J. E. Peralta, F. Ogliaro, M. J. Bearpark, J. J. Heyd, E. N. Brothers, K. N. Kudin, V. N. Staroverov, T. A. Keith, R. Kobayashi, J. Normand, K. Raghavachari, A. P. Rendell, J. C. Burant, S. S. Iyengar, J. Tomasi, M. Cossi, J. M. Millam, M. Klene, C. Adamo, R. Cammi, J. W. Ochterski, R. L. Martin, K. Morokuma, O. Farkas, J. B. Foresman, D. J. Fox, Wallingford, CT, 2016.

[2] T. Lu, F. Chen, *J. Comput. Chem.*, 2012, **33**, 580-592.

[3] T. Lu. *J. Chem. Phys.*, 2024, **161**, 082503.

[4] H. Sun, *J. Phys. Chem. B*, 1998, **102**, 7338-7364.

[5]. X. Yang, Z. Feng, M. Alshurafa, M. Yu, A. Foster, H. Zhai, T. Yuan, Y. Xiao, C. D'Agostino, L. Ai, M. Perez-Page, K. Smith, F. Foglia, A. Lovett, T. Miller, J. Chen, P. Budd and S. Holmes, *Adv. Mater.*, 2025, **37**, 2419534.

[6]. J. Zhang, B. Liu, H. Chen, T. Hu, S. Zhang, S. Chen, C. Zhao and X. Li, *J. Membr. Sci.*, 2026, **738**, 124843.

[7]. R. Lv, Z. Peng, Y. Wu, M. Gomaa and J. Yang, *J. Membr. Sci.*, 2026, **742**, 125120.

[8]. S. Nan, Y. Qiao, S. Qiu, S. Liu, T. Zhao, S. Ding and R. He, *J. Membr. Sci.*, 2026, **742**, 125127.

[9]. T. Yang, W. Jin, Y. Tang, X. Ye, W. Zhang, J. Li, H. Li, Y. Sun, Y. Dong, X. Zhang and C. Gong, J. Membr. Sci., 2025, **733**, 124325.

[10]. Y. Li, Z. Xu, W. Shi, M. Wang, Z. Lin, D. He, Y. Pan J. Liao, E. Ang and J. Shen, *J. Membr. Sci.*, 2025,**727**,124127.

[11]. Y. Jin, T. Wang, X. Che, J. Dong, Q. Li and J. Yang, *J. Power Sources*, 2022, **526**, 231131.

[12]. F. Arslan, K. Chuluunbandi, A. Freiberg, A. Kormanyos, F. Sit, S. Cherevko, J. Kerres, S. Thiele and T. Böhm, *ACS Appl. Mater. Interfaces*, 2021, **13**, 56584-56596.

[13]. H. Cho, E. Tas, J. Seiler, P. Atanasova and V. Atanasov, *J. Membr. Sci.*, 2026, **742**, 125091.

[14]. Q. Wang, L. Wang, M. Zhang, Z. Peng, Y. Lu, P. Lv and J. Yang, *Chem. Commun.*, 2024, **60**, 5318.

[15]. Q. Liu, Y. Luo, S. Yang, Y. Xiong, R. Wang, X. Fu, R. Zhang, S. Hu, X. Bao and C. Xu, *J. Power Sources*, 2023, **559**, 232666.

[16]. B. Liu, Y. Duan, T. Li, Y. Pang, Q. Liu, Q. Li, X. Hu and C. Zhao, *J. Membr. Sci.*, 2024, **692**, 122273.

[17]. Y. Shen, M. Liu, Z. Mao, T. Zhou, X. Han, Y. Song, Y. Wu, Y. Gao and W. He, *J. Membr. Sci.*, 2025, **731**, 124238.

[18. Q. Wang, S. Zhao, Y. Guo, W. Wei, L. Wang and J. Yang, *Sci. China Chem.*, 2025, **68**, 1078-1090.

[19]. T. Wang, Y. Jin, T. Mu, T. Wang and J. Yang, *J. Membr. Sci.*, 2022, **654**, 120539.

[20]. X. Zong, H. Mi, F. Chen, X. Guan, Y. Liu, W. Hu, N. Li, C. Jiang, Y. Lu, G. Zhu, W. Yan and J. Zhang, *Angew. Chem. Int. Ed.* 2025, **64**, e202509085.

[21]. C. Ge, H. Tang, R. Li, Q. Ju, T. Guo, H. Gao, Z. Lv, C. Niu, K. Geng and N. Li, *J. Membr. Sci.*, 2024, **692**, 122297.

[22]. P. Zhou, B. Zhang, L. Sang, H. Zhang, X. Sun, H. Han, Y. Chen, X. Li, J. Zheng, S. Li and S. Zhang, *J. Membr. Sci.*, 2025, **736**, 124643.

[23]. W. Li, J. Fang, Z. Mao, S. Lu and Y. Xiang, *J. Membr. Sci.*, 2025, 734, 124388.

[24]. D. Cai, T. Guo, G. Chao, X. Fu, Q. Liu, S. Hu, R. Zhang, K. Geng, N. Li, *J. Membr. Sci.*, 2026, **738**, 124773.

[25] P. Wang, Y. Wu, W. Lin and L. Wang, *J. Mater. Chem. A*, 2022, **10**, 23058.

[26] W. Wu, X. Guan, S. Zhang, G. Ma, C. Li, X. Zhou, D. Yu, Y. Luo, S. Wang, *J. Membr. Sci.*, 2025, **713**, 123383.

[27] X. Sun, H. Yu, J. Guan, B. Zhang, J. Zheng, S. Li and S. Zhang, *J. Mater. Chem. A*, 2024, **12**, 24499.
